# Artificial intelligence for predicting and preventing adverse pregnancy outcomes addressing bias and clinical translation

**DOI:** 10.3389/fdgth.2026.1841706

**Published:** 2026-06-19

**Authors:** Sharmake Gaiye Bashir, Hiba Abdi Salad, Yakub Burhan Abdullahi, Yusuf Hared Abdi, Mohamed Sharif Abdi, Naima Ibrahim Ahmed, Shuaibu Saidu Musa, Nafisa M. K. Elehamer, Muhammad Kabir Musa, Obasanjo Bolarinwa, Olusegun Dada

**Affiliations:** 1 Faculty of Health Sciences and Tropical Medicine, Somali National University, Mogadishu, Somalia; 2 Center for Health Research and Innovation, Somali National University, Mogadishu, Somalia; 3 Faculty of Health Science, Salaam University, Mogadishu, Somalia; 4 School of Global Health, Faculty of Medicine, Chulalongkorn University, Bangkok, Thailand; 5 Department of Nursing Science, Ahmadu Bello University, Zaria, Nigeria; 6 Department of Public Health and Epidemiology, Faculty of Medicine, University of Debrecen, Debrecen, Hungary; 7 Department of Health Education, Faculty of Public and Environmental Health, University of Khartoum, Khartoum, Sudan; 8 Department of Clinical Pharmacy, University of Michigan College of Pharmacy, Ann Arbor, MI, United States; 9 Department of Biomedical Sciences, Nazarbayev University School of Medicine, Astana, Kazakhstan; 10 Department of Global Healthcare Management, York St John University, London, United Kingdom; 11 Demography and Population Studies Programme, University of the Witwatersrand, Johannesburg, South Africa; 12 Department of Computer Science, University of Lagos, Nigeria

**Keywords:** artificial intelligence, bias, clinical translation, global health, health equity, machine learning, maternal health, pregnancy outcomes

## Abstract

Artificial intelligence (AI) has emerged as a promising approach for improving the early detection and management of adverse pregnancy outcomes through enhanced risk prediction and clinical decision support. This **narrative review** synthesizes current evidence on AI applications for predicting major obstetric complications, including preeclampsia, preterm birth, gestational diabetes, and fetal growth restriction. Reported predictive performance across studies demonstrates considerable heterogeneity, with area under the receiver operating characteristic curve (AUROC) values ranging from approximately 0.73 to 0.97, reflecting differences in datasets, model architectures, and validation strategies. Beyond predictive accuracy, this review critically examines sources of algorithmic bias that may influence model performance and equity in maternal healthcare. Eight key bias mechanisms are identified, including sampling bias, measurement bias, algorithmic bias, temporal bias, selection bias, labelling bias, deployment context bias, and access bias. These biases may limit model generalizability and risk amplifying existing maternal health disparities, particularly in low- and middle-income countries. Current evidence is further constrained by limited external validation across diverse populations, the absence of prospective clinical impact trials, insufficient cost-effectiveness analyses, and evolving regulatory frameworks governing AI accountability. The review discusses potential pathways for responsible clinical translation, emphasizing inclusive dataset development, rigorous multisite validation, careful integration into clinical workflows with human oversight, and strengthening regulatory and workforce capacity. Achieving equitable implementation of AI in maternal health will require deliberate efforts to embed transparency, accountability, and health equity throughout the AI development and deployment lifecycle.

## 1 Introduction

Maternal and perinatal mortality remain formidable global health challenges, with an estimated 287,000 maternal deaths occurring worldwide in 2020, 94% of which occur in low- and middle-income countries (1). Sub-Saharan Africa bears a disproportionate burden, accounting for nearly 70% of global maternal deaths, with maternal mortality rates exceeding 500 per 100,000 live births in several countries (2). Despite international commitments to reduce maternal mortality by three-quarters under the Sustainable Development Goals, progress remains insufficient, with many countries requiring a doubling of their current annual rate of reduction to meet the 2030 targets (3, 4). Postpartum hemorrhage, hypertensive disorders, sepsis, and complications from unsafe abortion continue to be the leading direct causes of maternal death, whereas preeclampsia, gestational diabetes, and preterm birth impose substantial morbidity burdens on both mothers and newborns (5).

Traditional risk assessment approaches for adverse pregnancy outcomes rely predominantly on demographic characteristics, medical history, and clinical parameters evaluated during discrete antenatal visits (6, 7). However, these conventional risk stratification tools demonstrate significant limitations in terms of sensitivity and specificity, with estimates ranging from 0% to 100% across different populations and conditions (6). The static nature of traditional risk assessment fails to capture the dynamic evolution of pregnancy complications, whereas subjective clinical judgment introduces inconsistencies between assessors (8, 9). Furthermore, existing models struggle to integrate the complexity and heterogeneity of multimodal data sources, which range from clinical biomarkers and ultrasound imaging to electronic health records and social determinants of health, thereby limiting their predictive accuracy and clinical utility (7, 10).

Artificial intelligence (AI) and machine learning technologies have emerged as promising tools to address these limitations, offering capabilities for pattern recognition in high-dimensional datasets, continuous risk monitoring, and personalized prediction (10, 11). Recent applications have demonstrated AI's potential across the spectrum of maternal healthcare, including early prediction of preeclampsia, gestational diabetes, preterm birth, and adverse neonatal outcomes, with reported predictive accuracies exceeding 85% in some studies (12, 13). Machine learning algorithms, such as random forest, extreme gradient boosting, and deep neural networks, have demonstrated promising predictive performance in several studies, although results vary across datasets and validation settings (14, 15). Time-lapse imaging analysis, natural language processing of clinical notes, and integration of multi-omics data further expand AI's capacity to phenotype pregnancies and stratify risk with unprecedented granularity (16).

However, enthusiasm for AI-driven maternal health solutions must be tempered by mounting concerns about algorithmic bias, health equity, explainability, and clinical readiness (17, 18). Most studies have revealed that AI models trained on homogeneous datasets perpetuate existing healthcare disparities, with algorithms demonstrating suboptimal performance for racial and ethnic minorities, socioeconomically disadvantaged populations, and underrepresented geographic regions (17, 19, 20). Additionally, the opaque nature of complex machine learning models poses challenges for clinical trust and shared decision-making, while inadequate validation, poor calibration across diverse populations, and limited transparency hinder translation from research settings to real-world implementation (21). Furthermore, ethical considerations about data privacy, algorithmic accountability, and the risk of automating discriminatory practices in maternal care require rigorous attention (22, 23).

This narrative review aims to synthesize current evidence on artificial intelligence applications for predicting adverse pregnancy outcomes, critically examine sources of algorithmic bias and methodological limitations, and explore pathways for responsible clinical translation. Therefore, we examined the sources of algorithmic bias in maternal health AI, assessed the adequacy of explainability approaches for obstetric decision support, and identified actionable pathways to ensure the equitable, trustworthy, and clinically meaningful implementation of AI technologies in maternal healthcare systems.

## 2 Methodology

### 2.1 Review design

This study was conducted as a structured narrative review to synthesize current evidence regarding the application of artificial intelligence (AI) for predicting and preventing adverse pregnancy outcomes, with particular emphasis on predictive performance, algorithmic bias, health equity implications, and pathways for responsible clinical translation. A narrative synthesis approach was selected because the literature in this field is methodologically heterogeneous and encompasses observational studies, machine learning model development studies, retrospective and prospective cohort analyses, systematic reviews, implementation studies, and conceptual or ethical frameworks. This methodological diversity limits the feasibility of quantitative pooling and formal meta-analysis. Therefore, a structured narrative approach was considered the most appropriate method to integrate diverse forms of evidence while enabling critical interpretation of methodological quality, translational relevance, and emerging implementation challenges in maternal healthcare.

### 2.2 Literature search strategy

A comprehensive literature search was conducted to identify peer-reviewed studies examining artificial intelligence applications in maternal and perinatal healthcare. Electronic databases including PubMed/MEDLINE, Scopus, Web of Science, and Google Scholar were systematically searched for articles published between January 2015 and December 2025. Additional studies were identified through manual screening of reference lists from eligible articles, narrative reviews, and systematic reviews relevant to AI-assisted maternal health prediction.

The search strategy combined controlled vocabulary terms and free-text keywords related to artificial intelligence, machine learning, maternal health, pregnancy complications, prediction modeling, and clinical implementation. Boolean operators (“AND” and “OR”) were used to optimize sensitivity and specificity of the search strategy across databases.

Representative search syntax included combinations such as:

(“artificial intelligence” OR “machine learning” OR “deep learning” OR “neural network*” OR “predictive algorithm*” OR “clinical decision support”) AND (“maternal health” OR “pregnancy outcome*” OR “obstetric complication*” OR “perinatal outcome*”) AND (“preeclampsia” OR “preterm birth” OR “gestational diabetes” OR “fetal growth restriction” OR “stillbirth” OR “postpartum hemorrhage”) AND (“prediction” OR “risk stratification” OR “screening” OR “early detection” OR “clinical implementation”).

Database-specific adaptations of search syntax and indexing terminology were applied where appropriate. The search process was designed to achieve broad thematic coverage of predictive AI applications, validation studies, ethical considerations, algorithmic bias, implementation barriers, and translational challenges relevant to maternal healthcare systems.

Because this review was conducted as a structured narrative synthesis rather than a formal systematic review, the search strategy prioritized conceptual breadth, methodological diversity, and critical thematic integration over exhaustive quantitative aggregation.

### 2.3 Eligibility criteria

Studies were considered eligible if they met one or more of the following inclusion criteria:

**Inclusion Criteria**

- Peer-reviewed studies reporting applications of artificial intelligence, machine learning, deep learning, or predictive analytics in maternal or perinatal health.
- Studies evaluating prediction or early detection of adverse pregnancy outcomes, including preeclampsia, preterm birth, gestational diabetes mellitus, fetal growth restriction, stillbirth, postpartum hemorrhage, or related obstetric complications.
- Studies reporting predictive performance metrics such as area under the receiver operating characteristic curve (AUROC), accuracy, sensitivity, specificity, calibration, or validation outcomes.
- Systematic reviews, methodological analyses, implementation studies, or conceptual papers discussing algorithmic bias, explainability, fairness, ethical considerations, equity implications, or clinical translation of AI in obstetric care.
- Studies examining AI-assisted clinical decision support systems, risk stratification tools, wearable monitoring technologies, or digital maternal health platforms.

**Exclusion Criteria**

- Studies unrelated to maternal, obstetric, fetal, or perinatal health outcomes.
- Conference abstracts, opinion papers, or editorials lacking sufficient methodological detail or analytical content.
- Non-English publications for which reliable translation was unavailable.
- Studies focused exclusively on non-clinical computational modeling without relevance to maternal healthcare applications.
- Articles lacking sufficient information regarding model methodology, validation, or clinical interpretation.

### 2.4 Study selection and evidence synthesis

Retrieved records were initially screened based on titles and abstracts to determine relevance to artificial intelligence applications in maternal health and adverse pregnancy outcomes. Potentially eligible articles subsequently underwent full-text review to assess conceptual relevance, methodological adequacy, and alignment with the objectives of the review. Study selection and thematic categorization were conducted through iterative discussion among the review authors to ensure consistency in interpretation and synthesis.

Rather than conducting a formal quantitative meta-analysis, findings were synthesized using a thematic narrative approach to accommodate substantial heterogeneity across study populations, data sources, machine learning architectures, outcome definitions, validation strategies, and implementation settings. This approach enabled integration of evidence spanning predictive modeling, clinical translation, ethical analysis, and health systems implementation.

The synthesis was organized into several major thematic domains:

1. Artificial intelligence applications for predicting specific pregnancy complications
2. Predictive performance and validation characteristics of existing AI models
3. Sources of algorithmic bias and implications for maternal health equity
4. Implementation barriers and health system challenges
5. Governance, regulatory, and ethical considerations
6. Pathways for safe, equitable, and clinically meaningful AI translation

Studies summarized in Table 1 and Table 2 partially overlapped but were synthesized according to their primary analytical focus. Table 1 primarily summarizes studies evaluating predictive AI applications, model performance, and validation characteristics for adverse pregnancy outcomes, whereas Table 2 synthesizes evidence related to bias mechanisms, ethical risks, equity implications, and implementation challenges associated with AI deployment in maternal healthcare settings.

**Table 1:** Key artificial intelligence applications for predicting adverse pregnancy outcomes.

Pregnancy outcome	Data inputs	AI modeling approach	Reported performance (AUROC)	Validation setting	Deployment stage
Preeclampsia (early-onset)	Mean arterial pressure, maternal BMI, obstetric history, PlGF, PAPP-A, sFlt-1, uterine artery Doppler, ECG parameters (24–27).	XGBoost, Random Forest, Neural Networks, Voting Classifier ensemble (24, 25, 28).	0.88–0.97 (24, 25, 28).	Multi-country external validation; prospective population cohorts (China, Spain, international PIERS) (24, 26).	Validation/experimental; limited clinical deployment (24, 26, 29).
Preeclampsia (term)	Maternal age, BMI, parity, prior PE, blood pressure, biochemical markers (PlGF, PAPP-A) (24).	Logistic Regression, Extra Trees Classifier, Stacking Classifier, Gaussian Process Classifier (24).	0.80–0.86 (24).	Population-based cohort with internal cross-validation; external validation limited (24, 48).	Experimental; research settings only (24, 49).
Preterm birth (<37 weeks)	Electronic health records, obstetric history, cervical length, biochemical markers, maternal demographics, prenatal care adequacy (30, 31).	Random Forest, Long Short-Term Memory (LSTM) deep learning, blended ensemble models (31, 32).	0.73–0.85 (31, 32).	Retrospective cohorts with internal validation; temporal validation sparse; limited external validation (31, 32).	Experimental; pilot implementation in select centers (31, 35).
Preterm birth (<32 weeks)	Maternal characteristics, amniotic fluid volume, gestational weight gain, prior preterm birth, household income (32–34).	Gradient Boosting, Random Forest, Support Vector Machines (33, 34).	0.85–0.92 (33, 34).	Retrospective single-center and multi-center cohorts; prospective validation limited (30, 33).	Experimental; research validation phase (32, 36).
Gestational diabetes mellitus	Maternal age, BMI, family history, prior GDM, fasting glucose, HbA1c, lipid profiles, first-trimester biomarkers (12, 38, 39).	XGBoost, CatBoost, LightGBM, Random Forest, AutoML (40, 41, 49).	0.75–0.95 (40, 41, 49).	Population-based cohorts with internal validation; limited external validation; mostly retrospective (39, 40).	Validation; some pilot screening programs in select institutions (49, 50).
Gestational diabetes (pharmacotherapy need)	Oral glucose tolerance test results, gestational week at diagnosis, early pregnancy metabolic markers (42, 43).	Logistic Regression, Gradient Boosting, Random Forest (42, 43).	0.75 (42).	Retrospective cohorts; population-based validation studies (42, 43).	Experimental; research phase (42, 43).
Fetal growth restriction	Fetal biometry (ultrasound), Doppler velocimetry (uterine/umbilical artery), fetal heart rate variability (CTG), maternal characteristics, placental biomarkers (44, 45).	Support Vector Machines, Neural Networks, Gradient Boosting, Convolutional Neural Networks for biometry (45, 46).	0.85–0.92 (accuracy: 97% for FHR-based models) (45, 46).	Systematic review/meta-analysis pooled data; retrospective single-center studies; prospective validation rare (45).	Experimental; not clinically deployed; requires algorithm refinement (44).
Postpartum hemorrhage	Maternal characteristics, obstetric history, laboratory parameters (hemoglobin, platelet count, aPTT), mode of delivery (43, 51).	Random Forest, XGBoost, Gradient Boosting, Logistic Regression with elastic-net (51, 52).	0.66–0.97 (51, 52).	Prospective cohorts with external validation; multi-center retrospective studies (43, 51).	Validation; experimental clinical trials; not routinely implemented (51, 52).
Emergency delivery in early-onset preeclampsia	Maternal characteristics, blood pressure, laboratory markers, proteinuria, gestational age at diagnosis (53).	Support Vector Machines with evolutionary feature selection (53).	0.79 (53).	Retrospective single-center; internal validation only (53).	Experimental; research phase (53).

Early-onset preeclampsia was defined as preeclampsia diagnosed before 34 weeks of gestation.

**Table 2:** Sources of bias and ethical risks in AI applications for maternal health.

Bias type	Source of bias	Clinical consequences	Populations most affected	Potential mitigation strategies	Reference
Sampling bias	Training datasets systematically underrepresent racial/ethnic minorities, low-income populations, rural communities, and women from LMICs due to differential healthcare access, exclusion from clinical trials, and geographic concentration of data collection in high-resource tertiary centers.	Lower sensitivity and specificity for predicting adverse outcomes in underrepresented populations leads to missed preventive interventions (aspirin, progesterone, cerclage) for truly high-risk women; false reassurance for minority women at elevated baseline risk; resource misallocation favoring already-privileged populations.	Racial and ethnic minorities (Black, Hispanic, Indigenous, Asian subgroups); low-income women; rural communities; pregnant women in Sub-Saharan Africa, South Asia, Latin America; refugees and migrants; women with limited English proficiency.	Intentional oversampling of underrepresented populations; federated learning across diverse geographic settings; mandatory reporting of dataset demographic composition; multi-site validation requiring representation from LMICs; community-based participatory research in algorithm development; synthetic data augmentation for minority groups.	(17, 70, 71, 82, 83)
Measurement bias	Medical devices and diagnostic tools calibrated predominantly on white European populations produce systematically different measurements for other racial groups (e.g., pulse oximetry overestimating oxygen saturation in Black patients); differential quality of imaging equipment and laboratory standards across settings.	Inaccurate risk stratification based on biased physiological measurements compounds health disparities; false classification of minority patients as low-risk despite elevated true risk; over-diagnosis and unnecessary interventions in populations where measurements are unreliable; erosion of clinical trust when algorithm recommendations conflict with clinical assessment.	Black and dark-skinned populations for pulse oximetry bias; Asian populations for body mass index-based risk prediction; communities served by under-resourced laboratories with older equipment; LMICs with inconsistent diagnostic standardization.	Race-stratified model development and validation; device-specific calibration curves for diverse populations; correction algorithms for known measurement biases; standardization of diagnostic protocols across settings; investment in high-quality diagnostic infrastructure in under-resourced regions; prospective validation studies examining measurement reliability across populations.	(21, 72, 84, 85)
Algorithmic bias	Learning algorithms amplify existing patterns in training data, encoding structural inequities as normative standards; optimization for overall accuracy prioritizes majority population performance at expense of minority groups; feature selection privileges biomarkers accessible primarily in well-resourced settings.	Differential performance across demographic groups results in unequal access to preventive care; minority women incorrectly stratified as low-risk miss timely aspirin prophylaxis or cervical surveillance; perpetuation of existing maternal mortality disparities through systematically less accurate predictions for Black, Hispanic, and Indigenous women.	Black women (3-4x higher maternal mortality despite algorithm underperformance); Hispanic women; Indigenous populations; low socioeconomic status groups; women in LMICs; communities with historical medical mistreatment.	Fairness-aware machine learning incorporating demographic parity, equalized odds, or predictive parity constraints; adversarial debiasing techniques; separate model development for distinct populations; ensemble methods combining population-specific models; mandatory fairness audits before deployment; algorithmic impact assessments.	(17, 21, 73–75, 82–84)
Temporal bias	Models trained on historical data reflect outdated clinical practices, treatment patterns, and disease epidemiology that do not represent current populations; secular trends in risk factor prevalence (obesity, hypertension) alter predictive relationships; changes in diagnostic criteria and treatment guidelines reduce model calibration over time.	Model performance degrades over time requiring continuous recalibration; predictions become unreliable as population risk profiles shift; interventions allocated based on outdated risk estimates; clinical decision support provides misleading recommendations when epidemiological context changes.	Emerging populations with changing risk profiles (e.g., increasing obesity prevalence in previously low-prevalence regions); women in transitioning healthcare systems; populations experiencing rapid epidemiological shifts; settings where treatment guidelines have recently changed.	Continuous model monitoring with drift detection; periodic recalibration using recent data; ensemble models incorporating temporal features; prospective validation studies; real-time performance tracking across demographic groups; automated alerts when model performance degrades; regular retraining schedules.	(21, 85, 86)
Selection bias	Differential healthcare-seeking behavior, insurance coverage, and geographic access to specialized obstetric care result in non-random sample selection; healthier patients more likely to attend routine antenatal visits contribute disproportionately to training data; severe cases transferred to tertiary centers overrepresented relative to community prevalence.	Algorithms reflect characteristics of women with healthcare access rather than population at large; underestimation of community-level risk when training data derived from facility-based cohorts; poor generalizability to underserved populations; exacerbation of disparities when AI tools deployed primarily in well-resourced settings.	Uninsured and underinsured women; geographically isolated rural populations; women with transportation barriers; undocumented immigrants; populations with limited healthcare access; women in conflict-affected regions.	Population-based sampling frames rather than convenience sampling; active recruitment of underserved populations; community outreach to ensure representative participation; weighting schemes to correct for selection probability; external validation in community-based cohorts; mandatory assessment of selection bias in model reporting.	(21, 87)
Label bias	Clinical outcome labels (e.g., preeclampsia diagnosis, indication for cesarean delivery) reflect implicit clinician biases in diagnostic workup intensity and management decisions; differential surveillance intensity causes ascertainment bias wherein complications detected more frequently in closely monitored populations.	Biased outcome labels perpetuate existing practice inequities wherein minority women receive differential care; algorithms learn to recommend less intensive surveillance or delayed intervention for populations historically undertreated; automation of discriminatory clinical decision-making patterns; reinforcement of implicit biases through apparent algorithmic objectivity.	Minority women receiving differential surveillance intensity; low-income women with less thorough diagnostic workups; populations in under-resourced facilities; women in settings with high implicit bias among providers.​	Standardized diagnostic criteria applied uniformly; blinded outcome adjudication; multiple independent reviewers for outcome classification; algorithmic bias audits examining outcome label patterns; explicit documentation of diagnostic uncertainty; incorporation of disagreement between clinicians as uncertainty measure.	(21, 75, 85)
Deployment context bias	Algorithms developed in high-resource academic medical centers with comprehensive EHRs, advanced imaging, and extensive laboratory panels require data inputs unavailable in community hospitals, rural clinics, and LMIC settings; implicit assumptions about infrastructure, staffing, and diagnostic capabilities embedded in model design.	AI tools designed for high-resource settings cannot be deployed in LMICs and rural areas where they are most needed; perpetuation of global health inequities; concentration of technological benefits in wealthy populations; inability to address global maternal mortality where burden is greatest.	Women in LMICs with limited specialist availability; rural communities without advanced imaging; settings with paper-based health records; health systems with inconsistent laboratory capacity; populations in humanitarian crises.	Development of resource-appropriate algorithms using minimal data inputs; tiered model architectures allowing degraded-but-functional performance with missing data; deployment feasibility assessments before implementation; co-design with end-users in target deployment settings; task-shifting strategies enabling use by frontline health workers; open-source models enabling local adaptation.	(62, 63, 75, 82, 83)
Access and digital divide bias	Unequal smartphone ownership, internet connectivity, digital literacy, and mobile health infrastructure limit accessibility of AI-enabled community screening tools in low-resource settings; language barriers and cultural acceptability of technology-mediated care reduce equitable deployment; cost barriers to wearable devices and home monitoring equipment.	Community-level screening tools remain inaccessible to populations with limited technology infrastructure; deepening of health disparities between connected and unconnected communities; exclusion of most vulnerable populations from preventive care innovations; widening gap between urban-wealthy and rural-poor maternal health outcomes.	Rural populations with limited broadband; low-income women unable to afford smartphones or data plans; elderly pregnant women with lower digital literacy; communities with limited electricity infrastructure; refugees in temporary settlements; women in countries with restrictive internet policies.	Offline-capable AI applications; SMS-based decision support not requiring smartphones; voice-based interfaces for low-literacy populations; subsidized device distribution programs; community health worker-mediated AI tools; partnerships with telecommunications providers for zero-rated health applications; culturally adapted interfaces; multilingual support.	(63–65)

Where available, predictive performance measures such as AUROC, sensitivity, specificity, calibration metrics, and validation context were extracted and interpreted alongside study design characteristics, including distinctions between internal validation, temporal validation, and external multicenter validation.

### 2.5 Consideration of evidence quality

Given the narrative nature of this review and the substantial methodological heterogeneity across included studies, a formal quantitative quality assessment or pooled risk-of-bias scoring framework was not performed. However, the review critically considered methodological characteristics relevant to the reliability, interpretability, and translational readiness of AI prediction models.

Key considerations included study design (retrospective vs. prospective), sample size adequacy, dataset representativeness, feature selection methodology, model overfitting risk, calibration performance, external validation status, transparency of reporting, and reproducibility of predictive findings. Particular attention was given to the distinction between internally validated models and studies demonstrating external or multicenter validation, as well as to the potential influence of sampling bias, measurement bias, and deployment-context bias on reported performance metrics.

This qualitative appraisal informed the interpretation of predictive accuracy claims, the discussion of algorithmic limitations, and the evaluation of real-world clinical applicability across diverse maternal healthcare settings, including low-resource and fragile health systems.

## 3 Current AI applications for predicting pregnancy risk

### 3.1 Hypertensive disorders of pregnancy

Machine learning models for preeclampsia prediction predominantly leverage maternal clinical characteristics, biochemical biomarkers, and biophysical measurements obtained during routine antenatal screenings (24, 25). First-trimester prediction models typically integrate mean arterial pressure, maternal age, body mass index, obstetric histo ry, and placental biomarkers, including placental growth factor, pregnancy-associated plasma protein-A, and soluble fms-like tyrosine kinase-1 (24, 26). More recent approaches have incorporated uterine artery Doppler pulsatility indices and electrocardiogram parameters to capture early cardiovascular adaptations that precede clinical manifestations (24, 27). Also, ensemble machine learning algorithms, particularly XGBoost and Random Forest, have demonstrated superior performance, with area under the receiver operating characteristic curves ranging from 0.84 to 0.973 across diverse populations (24, 28). The PIERS-ML model, validated across international cohorts, achieved robust discrimination for predicting adverse maternal outcomes in women already diagnosed with preeclampsia, with external validation confirming its generalizability (26). Early-onset preeclampsia prediction models utilizing gradient boosting decision trees and support vector machines have also reported predictive accuracies exceeding 90% when incorporating C-reactive protein, D-dimer, and hypoproteinemia alongside traditional clinical risk factors, although most findings have been derived from retrospective cohorts with limited external validation (28). Despite these promising metrics, clinical deployment remains limited, with most models confined to retrospective validation studies, rather than prospective implementation in routine prenatal care (24, 29).

### 3.2 Preterm birth

Artificial intelligence approaches to preterm birth prediction employ heterogeneous data sources spanning electronic health records, maternal demographic characteristics, obstetric history, cervical length measurements, and biochemical markers assessed throughout gestation (30, 31). Random Forest and Long Short-Term Memory deep learning architectures have emerged as at best effective, with the latter achieving an area under the curve value of 0.851 by capturing temporal dependencies in longitudinal clinical data (31). Blended ensemble models combining multiple classifiers have demonstrated accuracies of 73-75% with improved calibration compared to standalone algorithms (32). We now know that prediction performance varies substantially by preterm birth subtype, with iatrogenic preterm birth models consistently outperforming spontaneous preterm birth models across gestational age thresholds (33). Machine learning models predicting very preterm birth before 32 weeks achieved higher discrimination than models targeting any preterm birth before 37 weeks, reflecting stronger clinical signatures associated with more severe prematurity (33, 34). Key predictive features identified using explainable artificial intelligence techniques include abnormal amniotic fluid volume, adequacy of prenatal care, household income, prior preterm birth, and gestational weight gain trajectories (32, 35). Several studies have incorporated fetal heart rate variability patterns from cardiotocography, although external validation across different clinical settings remains limited (35, 36). The integration of wearable device data and continuous glucose monitoring represents an emerging frontier, although computational models utilizing these modalities require further refinement before their translation to clinical practice (37).

### 3.3 Gestational diabetes

Machine learning models for gestational diabetes prediction and management utilize preconception and early pregnancy risk factors, including body mass index (BMI), maternal age, family history of diabetes, prior gestational diabetes, and glycemic biomarkers measured before routine glucose tolerance testing (38, 39). CatBoost, LightGBM, and Random Forest algorithms have achieved area under the curve values of 0.75–0.84 when trained on maternal characteristics and first-trimester laboratory parameters, offering potential for risk stratification before the traditional 24–28 week screening window (40, 41). Explainable artificial intelligence frameworks employing SHAP values have identified fasting glucose, hemoglobin A1c, lipid profiles, and gestational weight gain as dominant predictors, aligning with the established pathophysiological mechanisms (12, 37). Beyond the prediction of gestational diabetes onset, machine learning models have been developed to forecast the need for pharmacological therapy vs. diet modification alone, with logistic regression and gradient boosting models achieving a median area under the curve of 0.75 (42). These treatment stratification models incorporate oral glucose tolerance test results, gestational age at diagnosis, and early pregnancy metabolic markers to guide individualized management approaches (42, 43). Preconception prediction models utilizing automated machine learning demonstrate promise for identifying high-risk individuals before conception, potentially enabling the development of primary prevention strategies (40). However, the majority of gestational diabetes prediction models exhibit limited external validation, and few have been prospectively evaluated in real-world clinical workflows to assess implementation feasibility and impact on maternal-fetal outcomes (39, 44).

### 3.4 Fetal growth restriction and stillbirth

Artificial intelligence applications for fetal growth restriction integrate ultrasound-derived fetal biometry, Doppler velocimetry indices, maternal characteristics, and placental biomarkers to identify high-risk pregnancies (44). Automated fetal biometry using convolutional neural networks has enhanced measurement precision and consistency compared to manual sonographic assessment, whereas machine learning models analyzing uterine and umbilical artery Doppler waveforms improve the prediction of placental insufficiency-related complications (44). Fetal heart rate variability extracted from cardiotocography represents the most frequently used input for intrauterine growth restriction prediction models, with support vector machines and neural networks achieving accuracies approaching 97% in retrospective cohorts (45). Recent gradient boosting and ensemble approaches combining biochemical markers, clinical risk factors, and ultrasound parameters have demonstrated area under the curve values of 0.85–0.92 for predicting adverse perinatal outcomes in pregnancies complicated by fetal growth restriction (46). Notably, artificial intelligence-based risk quantification has identified previously unrecognized high-risk clinical scenarios through complex interaction detection that exceeds the conventional risk stratification capabilities (46). Stillbirth prediction remains relatively understudied compared with other adverse pregnancy outcomes, with limited machine learning applications addressing this rare but catastrophic complication (47). The sparse literature on artificial intelligence for stillbirth prediction primarily focuses on identifying growth-restricted fetuses at elevated risk rather than population-wide screening approaches (47). The clinical translation of fetal growth restriction prediction models faces substantial challenges, including algorithm bias related to training dataset composition, insufficient standardization of diagnostic criteria across institutions, and ethical considerations regarding the balance between surveillance intensity and parental anxiety (44).

Table 1 presents artificial intelligence applications for predicting adverse pregnancy outcomes. Early-onset preeclampsia models achieved the highest performance (AUROC 0.88–0.97) with multi-country validation, while preterm birth models demonstrated an AUROC of 0.73–0.92, depending on the gestational age threshold. Gestational diabetes screening models report AUROC values ranging from 0.75 to 0.95, with emerging pilot implementation. Fetal growth restriction and postpartum haemorrhage models show promising accuracies (AUROC 0.85–0.97), although they remain in experimental phases. Despite robust performance metrics, most applications are currently in the validation or pilot testing stages, with limited routine clinical deployment. They require prospective external validation and workflow integration before they can be widely adopted.

## 4 Methodological quality and interpretation of predictive performance

Reported area under the receiver operating characteristic curve (AUROC) values for artificial intelligence models predicting adverse pregnancy outcomes demonstrate substantial heterogeneity, ranging from approximately 0.66 to 0.97 across studies. This variability likely reflects multiple methodological and clinical factors, including differences in dataset characteristics, study populations, feature selection strategies, outcome definitions, sample size, validation approaches, and model architectures, rather than indicating consistent superiority of any single algorithm across diverse clinical settings. Included studies employ diverse methodological approaches, ranging from traditional regression models to complex machine learning and deep learning algorithms, often using heterogeneous feature sets derived from clinical variables, imaging, or biomarker data. A substantial proportion of the evidence is derived from retrospective cohort studies using historical electronic health record datasets, which may introduce selection bias and limit generalizability to prospective clinical settings. In addition, many models rely primarily on internal validation techniques, whereas external validation in independent populations remains comparatively limited. Internal validation procedures such as cross-validation may overestimate predictive performance, particularly in studies with relatively small sample sizes, increasing the risk of overfitting and optimism bias. Furthermore, AUROC values alone provide limited insight into clinical usefulness unless accompanied by calibration assessment and clearly defined clinical decision thresholds. Consequently, reported predictive performance should be interpreted cautiously, and prospective multicenter validation studies remain essential to establish the real-world clinical utility and generalizability of artificial intelligence models in maternal healthcare.

**Figure 1:**
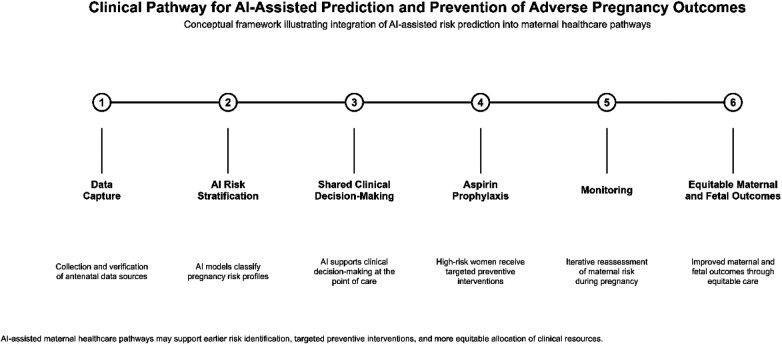
Clinical pathway for AI-assisted prediction and prevention of adverse pregnancy outcomes.

Figure 1 illustrates the conceptual workflow for integrating artificial intelligence into maternal healthcare decision-making. The process begins with the **data input stage**, where heterogeneous data sources including electronic health records, maternal demographic characteristics, laboratory biomarkers, ultrasound measurements, and obstetric history are collected. These inputs are processed during the **AI model development stage**, where machine learning algorithms analyze patterns and generate predictive models for adverse pregnancy outcomes. The model then produces **risk prediction outputs**, typically expressed as individualized probability scores for conditions such as preeclampsia, preterm birth, or gestational diabetes. These predictions are integrated into **clinical decision support systems**, where clinicians interpret the results alongside clinical judgment to guide surveillance strategies, preventive interventions, or referral decisions. Finally, a **feedback and monitoring stage** enables continuous model evaluation and updating through real-world clinical outcomes, improving model calibration, safety monitoring, and long-term performance.

## 5 Opportunities offered by AI in maternal health

Artificial intelligence has the potential to address longstanding challenges in maternal health through enhanced early detection, precision surveillance, and more efficient resource allocation across diverse healthcare settings (54, 55). The capacity for early risk identification represents perhaps the most clinically significant advantage, enabling intervention before complications manifest clinically (44, 56). Traditional antenatal screening relies on the detection of overt clinical signs that often emerge only after pathophysiological processes are well established, whereas emerging AI-driven prediction models may enable earlier identification of at-risk pregnancies by recognizing subtle pattern signatures in multimodal data (44). However, much of the current evidence remains derived from retrospective datasets with limited prospective clinical validation. This temporal advantage is particularly valuable for conditions such as preeclampsia, where initiation of low-dose aspirin prophylaxis before 16 weeks of gestation achieves maximal efficacy; however, current risk stratification tools frequently fail to identify high-risk women sufficiently early in pregnancy (57, 58). Similarly, early identification of women with elevated preterm birth risk enables timely cervical length surveillance and preventive interventions during the optimal therapeutic window when progesterone supplementation or cervical cerclage demonstrates the greatest benefit (59). If prospectively validated and appropriately implemented, AI-assisted risk prediction could support a shift from reactive management of established complications toward more proactive prevention strategies in maternal healthcare (55, 56).

Beyond potential temporal advantages, AI-based approaches may support more individualized antenatal surveillance strategies based on continuously updated risk profiles rather than population-level averages or static categorical classifications (18, 56). Current guidelines apply uniform surveillance schedules determined primarily by gestational age and broad risk categories, resulting in both over-monitoring of low-risk pregnancies with attendant anxiety and healthcare costs and under-monitoring of high-risk pregnancies that might benefit from intensified assessment (10, 60). Machine learning algorithms may facilitate dynamic risk stratification by integrating evolving clinical information throughout pregnancy to support surveillance decisions and identify women who may benefit from intensified monitoring (44, 61). However, evidence supporting the safety and effectiveness of AI-guided surveillance strategies in routine clinical practice remains limited (44, 61). This personalized approach optimizes the balance between detection sensitivity and resource utilization, ensuring that clinical attention is concentrated where it will yield the greatest benefit (18, 56). The integration of wearable devices and remote monitoring technologies further enhances precision surveillance by capturing continuous physiological data, including blood pressure, heart rate variability, physical activity, and sleep patterns, which provide richer phenotypic characterization than intermittent clinic-based assessments and are particularly valuable for detecting gradual deterioration that might escape detection during discrete appointments (54).

In resource-constrained health systems, where specialist obstetric expertise and advanced diagnostic infrastructure are scarce, AI-augmented clinical decision support offers particular promise for enhancing frontline healthcare capacity (62, 63). Mobile health applications incorporating simplified risk assessment algorithms can empower primary care providers, midwives, and community health workers to conduct evidence-based risk stratification and make appropriate referral decisions without requiring specialist consultations for every case (61, 64). Field trials in sub-Saharan Africa have demonstrated that mobile health clinical decision support systems delivering emergency obstetric protocols via text messaging and unstructured supplementary service data significantly improved frontline health workers' adherence to evidence-based guidelines and increased timely referral of high-risk cases to facility-based care (64, 65). These digital tools may help extend specialist decision support to remote settings where access to maternal-fetal medicine expertise is limited, although their effectiveness and scalability remain dependent on infrastructure, workforce capacity, and local implementation contexts (62, 66). Furthermore, by automating routine data capture and generating real-time dashboards of facility-level maternal health indicators, AI-enabled systems reduce the documentation burden on clinical staff, while simultaneously strengthening health information systems that are essential for quality improvement and resource planning.

Artificial intelligence-based risk stratification tools may support more efficient allocation of limited healthcare resources by identifying women who may require tertiary referral, inpatient admission, or specialist consultation (18, 61). Prediction models that accurately discriminate between women at low vs. high risk for progression to severe complications enable healthcare systems to concentrate intensive monitoring, specialist consultations, and inpatient beds on those most likely to benefit while safely deferring resource-intensive interventions for lower-risk individuals (28, 53). This triage function becomes particularly valuable in low-resource contexts, where healthcare infrastructure cannot accommodate universal high-intensity surveillance, necessitating evidence-based prioritization to maximize population health outcomes within budget constraints (62, 63). Preliminary cost-effectiveness analyses suggest that AI-guided risk stratification may improve resource utilization and potentially reduce healthcare expenditures; however, robust prospective economic evaluations across diverse healthcare settings remain limited (67).

The potential of community-level screening tools represents an emerging frontier that could democratize access to sophisticated risk assessments (61, 68). Smartphone-based applications that analyze self-reported symptoms, home blood pressure measurements, and photographic images enable women to conduct preliminary risk assessments without requiring facility visits, with automated alerts prompting healthcare-seeking when concerning patterns emerge (61, 69). Proof-of-concept studies have explored artificial intelligence analysis of self-collected biological samples, including urine dipstick photographs for proteinuria detection and capillary blood glucose from home glucometers for gestational diabetes screening, potentially extending reach to geographically isolated populations or during public health emergencies that restrict facility access (61, 69). Natural language processing chatbots providing health education and symptom triage could further augment community-level capacity by answering common pregnancy-related questions, reinforcing medication adherence, and identifying red-flag symptoms that require urgent evaluation (63, 68). However, realizing this potential requires careful attention to digital literacy, device accessibility, connectivity infrastructure, and integration with formal healthcare systems to ensure that community screening tools complement, rather than fragment, care delivery (64, 65). Despite these promising opportunities, relatively few AI-based maternal health tools have undergone prospective clinical implementation trials evaluating their impact on maternal or perinatal outcomes in real-world healthcare settings.

## 6 Bias, equity, and ethical challenges

The promise of artificial intelligence in maternal health confronts formidable challenges rooted in data bias, algorithmic inequity, and structural barriers that threaten to perpetuate or amplify existing disparities rather than ameliorate them (17, 70). Data representativeness is perhaps the most fundamental source of algorithmic bias, with training datasets systematically underrepresenting racial and ethnic minorities, low-income populations, and women from low-income and middle-income countries (70, 71). A systematic examination of electronic health records revealed that inherent biases arise from multiple sources, including differential healthcare access patterns, implicit clinician biases encoded in clinical documentation, measurement artifacts from medical devices calibrated predominantly on white populations, and the systematic exclusion of marginalized groups from clinical research that generates labeled training data (72). Pregnancy prediction models trained predominantly on well-resourced tertiary centers in high-income countries capture clinical signatures reflective of those specific populations and settings but may fail to generalize to under-resourced facilities, rural populations, or countries with different disease epidemiology and healthcare delivery structures (19, 70). The cascade effect of these data limitations manifests as algorithms that demonstrate robust performance in validation cohorts resembling training populations, but exhibit substantial performance degradation when deployed in demographically or geographically distinct settings, precisely the populations that might benefit most from AI-augmented decision support (70, 73).

Algorithmic bias translates data representativeness failures into differential clinical performance across demographic groups, with mounting evidence documenting that prediction models achieve lower sensitivity, specificity, and positive predictive values for racial and ethnic minorities than for majority populations (17, 71). A scoping review examining racial and ethnic bias in artificial intelligence health algorithms between 2020 and 2024 found that models regularly outperformed humans in diagnostic precision for majority populations, while simultaneously exhibiting suboptimal performance for Black, Hispanic, and Asian individuals, effectively encoding structural racism into ostensibly objective computational tools (17, 73). In obstetric applications, this manifests as preeclampsia prediction models with lower area under the receiver operating characteristic curve values for black women despite their elevated baseline risk, preterm birth algorithms that misclassify Hispanic women at higher rates, and gestational diabetes screening tools calibrated primarily for Asian populations that demonstrate poor transferability to other racial groups (48, 74). The implications extend beyond statistical measures to tangible clinical consequences, as women from underrepresented groups receive less accurate risk stratification, potentially missing preventive interventions when falsely categorized as low-risk, or experiencing unnecessary surveillance and anxiety when incorrectly flagged as high-risk (70, 71). These algorithmic failures occur against a backdrop of existing maternal health disparities, wherein black women in the United States face maternal mortality rates three to four times higher than white women, suggesting that biased AI systems deployed without rigorous equity evaluation could further entrench rather than reduce longstanding inequities (17, 75).

Structural inequities in healthcare access, quality, and delivery fundamentally limit AI generalizability across contexts that differ substantially from those in algorithm development settings (63, 75). The dominant paradigm of developing sophisticated machine learning models in well-resourced academic medical centers using comprehensive electronic health records, advanced imaging modalities, and extensive laboratory panels creates the implicit assumption that a similar infrastructure exists wherever algorithms will be deployed (62, 75).

### 6.1 Implementation challenges and governance barriers in low-resource settings

However, the majority of global maternal deaths occur in settings characterized by limited specialist availability, inconsistent ultrasound access, intermittent laboratory capacity, and paper-based or rudimentary electronic documentation systems that capture only a fraction of the data inputs required by high-performing algorithms (62, 63). This infrastructure mismatch renders many promising AI applications essentially non-transferable to settings where they might deliver the greatest public health impact, perpetuating a digital divide wherein technological advances widen rather than narrow global health disparities (70, 76). Furthermore, structural inequities manifest in differential quality of care that becomes encoded in training data, wherein minority patients and those from disadvantaged socioeconomic backgrounds may receive less thorough diagnostic workups, experience longer delays in specialist referral, and have suboptimal management of complications patterns that algorithms learn to replicate as “standard care” rather than recognize as disparities requiring correction (75).

#### 6.1.1 Clinical integration and decision thresholds

For AI prediction models to generate meaningful clinical benefit, their outputs must be integrated into clearly defined clinical decision pathways. In obstetric practice, predicted risk scores should be linked to actionable thresholds that trigger specific interventions, such as intensified antenatal monitoring, early referral to specialist care, initiation of prophylactic therapies, or delivery planning in high-risk facilities. Importantly, algorithmic predictions should complement rather than replace clinical judgment, with clinicians interpreting AI-generated risk estimates within the broader clinical context. False positive predictions may increase surveillance intensity, whereas false negatives highlight the need for continuous model evaluation and safety monitoring. Emerging implementation studies suggest that the greatest clinical benefit occurs when AI systems are embedded within electronic decision support tools that provide clear recommendations alongside risk estimates, thereby supporting clinicians in translating predictive analytics into timely preventive action.

The “black box” nature of complex machine learning architectures poses profound challenges for clinical trust and adoption, particularly when algorithmic reasoning remains opaque to clinicians responsible for patient care decisions (77, 78). Systematic reviews examining the impact of explainable artificial intelligence on clinicians' trust paradoxically reveal that explanation provision can either increase or decrease trust, depending on explanation quality, the degree of alignment between algorithmic and clinical reasoning, and whether explanations expose model limitations or uncertainties that clinicians find disconcerting (78). Obstetricians trained in pathophysiological reasoning and pattern recognition developed through years of clinical experience may be reluctant to accept AI recommendations when the underlying logic remains inscrutable, particularly for high-stakes decisions such as the timing of delivery or escalation to cesarean section (78). The tension between algorithmic accuracy and interpretability creates a dilemma wherein the most performant models deep neural networks and ensemble methods offer the least transparency, while more interpretable approaches such as logistic regression sacrifice predictive power for comprehensibility (77). Current explainability techniques, including SHapley Additive exPlanations and Local Interpretable Model-agnostic Explanations, provide *post-hoc* rationalizations of model predictions, but these explanations may not align with clinical reasoning frameworks, may oversimplify complex multivariate interactions, or may generate inconsistent explanations across similar cases, ultimately failing to bridge the communication gap between algorithmic and clinical logic (78, 79).

Accountability and medicolegal concerns surrounding AI-augmented maternal care remain fundamentally unresolved, creating uncertainty regarding liability allocation when algorithms contribute to adverse outcomes (80, 81). The traditional medico-legal framework assigns responsibility to identifiable human actors the attending physician, consulting specialist, or institutional protocol committee but artificial intelligence introduces a distributed agency wherein multiple stakeholders participate in algorithm development, validation, deployment, and ongoing maintenance (75, 81). When an AI prediction model fails to identify a woman at high risk for preeclampsia who subsequently experiences eclamptic seizure or incorrectly stratifies a preterm birth risk leading to inadequate surveillance, determining whether liability rests with the algorithm developers, the healthcare institution that deployed the system, the clinician who accepted the recommendation, or the regulatory body that approved the device remains legally ambiguous (80, 81). This ambiguity may paradoxically incentivize defensive medicine wherein clinicians override AI recommendations even when algorithmically correct, negating the intended benefits of decision support, or conversely, create automation bias wherein clinicians defer excessively to algorithmic output, abdicating professional judgment in ways that compromise patient safety (78). The absence of clear regulatory frameworks governing AI liability, coupled with insufficient standards for validation rigor, post-market surveillance, and algorithmic transparency, leaves both clinicians and patients vulnerable to harm that the current legal structures struggle to adjudicate (75, 80). Fundamentally, the question of who bears responsibility when AI errors remain unanswered, creating a governance vacuum that impedes responsible clinical translation, even as technological capabilities advance (76, 81).

Table 2 systematically maps eight sources of bias and ethical risks across the AI development and deployment pathways for maternal health, documenting clinical consequences, vulnerable populations, and evidence-based mitigation strategies for each bias type. The table demonstrates that addressing bias and equity requires multifaceted interventions, spanning data collection, algorithm design, deployment strategy, and regulatory governance.

**Figure 2:**
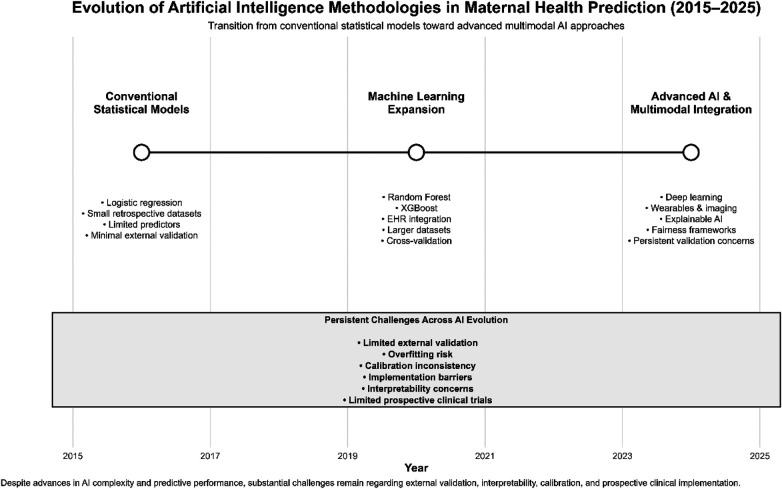
Evolution of artificial intelligence methodologies in maternal health prediction (2015–2025).

As illustrated in Figure 2, the rapid evolution of artificial intelligence methodologies between 2015 and 2025 has contributed substantially to methodological heterogeneity in maternal health models, from early logistic regression to recent ensemble and deep learning approaches using multimodal data, complicating direct AUROC comparisons (0.70–0.98) (88). While newer models often show higher internal accuracy, they suffer from limited interpretability and, critically, limited external validation, with only ∼39% tested on independent cohorts and external performance frequently dropping (e.g., sensitivity from 0.92 to 0.68) (89, 90). Variability in computational capacity, feature engineering, dataset sizes, and validation strategies further obscures cross-study comparisons, and despite emerging explainable AI and fairness frameworks, inconsistent outcome definitions and calibration approaches persist (91). Thus, higher reported AUROC values may not translate into real-world clinical effectiveness or generalizability, warranting cautious interpretation of AI's readiness for clinical translation.

## 7 Evidence gaps and implementation barriers

The translation of artificial intelligence from research prototypes to routine clinical deployment confronts substantial evidence gaps that extend beyond the ethical concerns addressed previously to encompass the fundamental questions of external validity, clinical impact, economic value, and regulatory preparedness (92, 93). Most critically, the systematic underrepresentation of low- and middle-income country populations in training datasets creates a circular evidence deficit wherein the settings bearing the greatest maternal mortality burden remain excluded from algorithm development, precluding validation in precisely the contexts where AI could deliver maximum public health benefits (94, 95). Systematic reviews examining AI applications for maternal health revealed that fewer than 15% of prediction models undergo external validation in populations distinct from development cohorts, and validation in low-resource settings remains very rare despite these regions experiencing 94% of global maternal deaths (95, 96). This geographic bias creates algorithms optimized for clinical workflows, diagnostic infrastructure, and disease epidemiology characteristics of high-income tertiary centers with uncertain or degraded performance when deployed in community hospitals, rural clinics, or facilities with limited laboratory capacity that define healthcare delivery for most pregnant women globally (95, 96). Infrastructure mismatch extends beyond data inputs to encompass fundamental assumptions about Internet connectivity, electronic health record interoperability, and technical support capacity that render many AI tools essentially non-deployable in resource-constrained settings (93).

Equally problematic is the paucity of prospective clinical impact studies that demonstrate whether AI-augmented care actually improves maternal and fetal outcomes compared with standard management (92, 93). The overwhelming majority of published AI research on maternal health reports retrospective algorithm development and internal validation using historical datasets, establishing statistical associations rather than demonstrating causality or clinical benefits (92, 93). Prospective trials randomizing women or healthcare facilities to AI-guided vs. conventional management remain scarce, leaving fundamental questions unanswered regarding whether improved risk prediction translates to reduced adverse outcomes or merely shifts resource allocation without changing population-level mortality (93). This evidence gap becomes particularly acute when considering potential harms, including false reassurance from incorrect low-risk classifications, anxiety and unnecessary interventions from false-positive high-risk predictions, and unintended consequences of changing clinical workflows in ways that might compromise holistic assessments (92). The absence of implementation science research examining how clinicians interact with AI recommendations in real-world practice creates uncertainty about whether algorithms will be used as intended, whether clinician override patterns introduce new biases, and whether automation bias might erode clinical reasoning skills over time (93, 97).

Cost-effectiveness analyses providing economic justification for AI investment remain limited and methodologically inconsistent, with most published economic evaluations relying on modeled projections rather than empirical cost data from actual deployments (98). The substantial upfront investment required for AI infrastructure, including algorithm licensing fees, electronic health record integration, clinician training, ongoing technical support, and continuous model retraining, must be weighed against potential savings from complication prevention and resource optimization, yet rigorous economic analyses comparing the total cost of ownership to health outcomes achieved are largely absent from the maternal health literature (67, 98). Particularly for low- and middle-income countries operating under severe budget constraints, the opportunity cost of investing in AI vs. strengthening fundamental healthcare infrastructure, training additional midwives and obstetricians, and improving medication supply chains remains unquantified (95, 96).

Regulatory frameworks governing AI medical devices demonstrate gaps and inconsistencies that impede responsible deployment, while failing to ensure adequate safety and efficacy standards (92, 99). Current regulatory pathways developed for traditional medical devices are poorly suited to continuous learning algorithms that evolve after deployment, software distributed as mobile applications rather than hardware, and clinical decision support tools occupying an ambiguous zone between medical devices and clinical information systems (92, 93). The absence of mandatory prospective validation requirements, standardized performance metrics across diverse populations, and post-market surveillance obligations creates a governance vacuum wherein algorithms can reach clinical use without demonstrating equivalent performance across demographic groups or documenting real-world safety in heterogeneous populations (21). Furthermore, regulatory fragmentation across jurisdictions creates barriers to international deployment, with each country requiring separate approval processes that prove particularly burdensome in low-resource settings lacking dedicated regulatory agencies with AI expertise (94, 95). These evidence and governance gaps collectively impede the responsible translation of AI from promising research tools to routine clinical practice, requiring coordinated action across research methodologies, health economics, regulatory science, and implementation research to bridge the gap between algorithmic potential and clinical reality (92, 93).

## 8 Pathways to safe and equitable clinical translation

Achieving an equitable translation of artificial intelligence for maternal health requires coordinated interventions across data infrastructure, validation frameworks, clinical integration, governance mechanisms, and workforce development that directly address the systematic failures documented in the preceding sections (76). The foundation rests on the intentional construction of inclusive datasets that transcend convenience sampling from well-resourced tertiary centers to actively recruit underrepresented populations through federated learning architectures, thereby enabling collaborative model training across geographically diverse sites without centralizing sensitive data (76, 100). Implementation of mandatory demographic reporting requirements specifying the racial, ethnic, socioeconomic, and geographic composition of training and validation cohorts would create accountability mechanisms that ensure that algorithm development proceeds with explicit attention to representativeness rather than treating equity as an afterthought (72). Community-based participatory research approaches that engage pregnant women from marginalized communities as active collaborators rather than passive data sources can identify culturally relevant risk factors, acceptable intervention modalities, and implementation barriers that are invisible to academic researchers, simultaneously strengthening the dataset quality and building trust essential for deployment acceptance (101). Synthetic data augmentation techniques, which generate statistically representative minority population samples, are methodologically complex and require careful validation to avoid introducing spurious associations and offer pragmatic pathways to balance severely imbalanced datasets when prospective recruitment is insufficient (83).

External validation constitutes an indispensable bridge from algorithmic promise to clinical reliability; however, current practice falls catastrophically short of the rigorous multi-site, multi-population evaluation essential for maternal health applications (95, 102). Establishing international validation consortia coordinating prospective data collection across high-, middle-, and low-income countries would enable the systematic assessment of algorithmic performance across the epidemiological, infrastructure, and demographic diversity characterizing global maternal healthcare (103, 104). Validation protocols must extend beyond discrimination metrics to encompass calibration across demographic subgroups, ensuring that predicted probabilities align with observed event rates for racial and ethnic minorities, rather than merely achieving an acceptable area under the curve values in aggregate populations (21, 104). Transparent reporting of subgroup-specific performance using standardized metrics and disaggregated demographic stratification exposes differential accuracy patterns currently obscured by aggregate statistics, enabling informed deployment decisions and triggering recalibration when equity thresholds are violated (102, 105). Prospective randomized implementation trials comparing AI-augmented vs. standard antenatal care represent the gold standard for establishing clinical benefits, moving beyond surrogate outcomes of risk classification accuracy to demonstrate actual reductions in maternal mortality, preeclampsia incidence, preterm birth rates, and other patient-centered endpoints (93, 102).

Clinical workflow integration requires meticulous attention to human factors, cognitive psychology, and implementation science to ensure that AI recommendations are enhanced rather than disrupted clinical reasoning and maintain patient-centered care as the organizing principle (97, 106). Decision support interfaces must present risk estimates with appropriate uncertainty quantification, explainable rationales highlighting which clinical features drove predictions, and actionable recommendations aligned with evidence-based interventions, rather than merely flagging high-risk status without guidance (78, 107). Structured override mechanisms capturing clinician rationale when rejecting AI recommendations create feedback loops for continuous model improvement while preventing automation bias, wherein clinicians defer excessively to algorithmic output (97, 102). Phased implementation begins with shadow deployment, wherein algorithms generate recommendations that clinicians can view but are not obligated to follow, allowing prospective monitoring of real-world performance before full integration, identifying calibration drift, unexpected failure modes, and workflow friction points requiring remediation (97, 102). Task-shifting strategies enabling midwives, community health workers, and primary care providers to operationalize AI-guided risk stratification extend specialist expertise to under-resourced settings, although success requires tailored training, technical support infrastructure, and sustainable financing mechanisms (62, 63).

Regulatory and ethical governance frameworks must evolve beyond device-centric paradigms developed for static medical equipment to address the unique challenges posed by continuous learning algorithms, software-as-medical-device platforms, and decision support systems straddling clinical and administrative functions (99). Mandatory fairness audits examining algorithmic performance across demographic groups before market authorization, coupled with ongoing post-deployment surveillance to detect performance degradation or emergent bias patterns, would establish accountability absent from current regulatory structures (76). Standardized reporting requirements modeled on TRIPOD + AI and CONSORT-AI guidelines should mandate the disclosure of dataset composition, validation methodology, subgroup performance metrics, and limitations, enabling the independent assessment of bias risk and appropriate use constraints (105). International regulatory harmonization through frameworks such as those proposed by the World could reduce fragmentation barriers while establishing minimum safety and equity standards applicable across diverse health system contexts (95, 108). Ethical review processes must incorporate community representatives, bioethicists, and health equity experts alongside technical reviewers to evaluate not only algorithmic accuracy but also broader implications for justice, autonomy, and equitable healthcare access (76, 107).

Workforce capacity development has emerged as the rate-limiting factor for sustainable AI implementation, requiring investment in training curricula, technical infrastructure, and inter-professional collaboration models that equip clinicians, data scientists, and health administrators with complementary competencies (108, 109). Medical and midwifery education must integrate AI literacy, encompassing algorithm limitations, bias recognition, appropriate skepticism toward black-box recommendations, and effective communication of risk estimates to diverse patient populations (104, 109). Conversely, data scientists developing maternal health algorithms require immersion in clinical contexts, obstetric physiology, and social determinants of health to encode appropriate causal assumptions, select clinically meaningful features, and recognize when algorithmic recommendations conflict with physiological plausibilities (76, 101). Establishing multidisciplinary implementation teams comprising obstetricians, midwives, informaticians, biostatisticians, and community health representatives creates the collaborative infrastructure necessary to navigate the sociotechnical complexity of AI deployment while maintaining patient welfare as the paramount objective (100, 107). These coordinated strategies collectively chart actionable pathways from the current landscape of promising but inequitable prototypes toward a future in which AI genuinely enhances maternal health outcomes across all populations and settings (76).

## 9 Patient perspectives, acceptability, and digital equity

Patient perspectives regarding AI-assisted maternal risk prediction remain comparatively underexplored, with emerging evidence suggesting mixed acceptability and important concerns regarding trust, transparency, and equity (110). While some pregnant women may value the potential for earlier risk identification and improved access to care, many continue to prefer clinician-led risk communication because of concerns that artificial intelligence could depersonalize care and weaken the therapeutic clinician-patient relationship (111). Additional concerns include algorithmic opacity, confidentiality of maternal health data, and the possibility that AI-generated risk classifications may increase anxiety or contribute to stigmatization if communicated without appropriate counseling and contextual interpretation (112). In low-resource settings, digital literacy barriers, unequal access to smartphones, and inconsistent internet connectivity may further exacerbate existing maternal health inequities, particularly where AI systems assume reliable digital infrastructure and technological familiarity (110). Sociocultural and linguistic factors may also influence acceptance of AI-assisted maternal healthcare, as algorithmic tools often lack adaptation to diverse languages, health beliefs, and local healthcare contexts (20). Importantly, AI-assisted decision support should complement rather than replace clinician-patient communication and shared decision-making processes (112). In addition, informed consent challenges remain substantial, particularly regarding secondary use of maternal health data, communication of algorithmic uncertainty, and explanation of potential biases embedded within predictive models (113). Overall, robust qualitative and implementation research evaluating long-term patient trust, engagement, and perceived fairness of AI-supported maternal healthcare remains limited, representing an important priority for equitable clinical translation.

## 10 Future opportunities and implementation pathways in low-resource settings

The distinctive healthcare challenges of low- and middle-income countries demand AI deployment models that are fundamentally different from those developed in affluent tertiary centers, creating opportunities for pragmatic innovation that leapfrogs infrastructure limitations rather than replicating high-resource paradigms (94, 114). Mobile-based decision support systems operating through SMS messaging, voice interfaces, and smartphone applications designed for offline functionality represent the most feasible near-term pathway, enabling community health workers, midwives, and primary care providers to access algorithmic risk stratification without requiring advanced electronic health records or broadband Internet connectivity (69, 115). Field implementations in Sub-Saharan Africa and South Asia demonstrate that SMS-based clinical alerts substantially improve adherence to evidence-based management protocols and reduce delays in the referral of high-risk cases to facility-based care, extending specialist expertise to geographically remote areas where conventional access to maternal-fetal medicine remains impossible (64, 116). Point-of-care ultrasound combined with artificial intelligence image analysis represents another high-impact opportunity, as portable ultrasound devices paired with algorithms for analyzing fetal biometry, placental adequacy, and amniotic fluid volume can function in basic clinics and community health posts lacking radiology expertise (115, 117). Critically, successful mobile health implementations in LMICs have demonstrated that technology deployment alone fails without parallel investment in training, technical support, regulatory clarity, and integration into existing health systems lessons applicable to AI translation that require concurrent attention to human factors and implementation science (118).

Emerging evidence has illuminated the intersection between climate change and maternal health, creating new opportunities for integrated early warning and surveillance systems that simultaneously address environmental monitoring and pregnancy outcomes (118). Climate drivers, including extreme heat exposure, air pollution, flooding, and drought, directly increase the risk of preterm birth, low birth weight, stillbirth, preeclampsia, gestational diabetes, and maternal death, with pregnant women experiencing particular vulnerability during the critical windows of gestation (119, 120). Artificial intelligence-powered climate-health surveillance systems can integrate real-time meteorological data, environmental sensors, and health facility reporting to generate facility-specific alerts when environmental conditions reach thresholds associated with adverse pregnancy outcomes, enabling proactive maternal health interventions even before women present clinical symptoms. Community-level screening programs that leverage mobile phones for maternal symptom reporting, home blood pressure measurement, and urine dipstick analysis create scalable pathways to detect warning signs of preeclampsia, gestational diabetes, and other complications in settings where routine antenatal visits remain infrequent or inaccessible (121). Data from health and demographic surveillance systems, when analyzed using artificial intelligence techniques, can identify emerging threats to maternal and neonatal health, including pathogen emergence, adverse environmental exposures, or healthcare system disruptions, enabling rapid public health responses before complications cascade into preventable deaths (122). The pragmatic approach to AI deployment in LMICs emphasizes high-impact problems, where evidence clearly demonstrates that improved risk identification translates to deliverable interventions within existing health system capacity, while building local expertise, data infrastructure, and sustainable financing mechanisms that enable long-term system strengthening rather than perpetuating technology dependence on external resources or expertise (94, 123).

## 11 Future directions

The evolution of artificial intelligence for maternal health will increasingly harness multi-omics integration, combining genomic, transcriptomic, proteomic, metabolomic, and microbiome data to construct comprehensive molecular signatures that capture the biological complexity underlying adverse pregnancy outcomes (16, 124). Proteomics-based prediction of preterm birth and preeclampsia has demonstrated that composite biosignatures outperform single biomarker approaches, whereas metabolomic profiling reveals dynamic metabolic shifts throughout gestation that precede clinical manifestations of complications by weeks or months (125). Deep learning architectures capable of integrating heterogeneous multi-omics datasets with clinical variables, imaging data, and wearable biosensor streams promise unprecedented precision in risk stratification, moving beyond population-level averages to truly personalized predictions that account for individual biological variation (124, 126). Wearable biosensors that enable continuous monitoring of maternal vital signs, fetal heart rate variability, physical activity patterns, and biochemical markers from interstitial fluid represent the next frontier for real-time pregnancy surveillance, shifting from episodic clinic-based assessment to longitudinal phenotyping that captures gradual deterioration invisible to discrete measurements (127, 128). The PowerMom platform and similar digital health initiatives demonstrate the feasibility of collecting multimodal data, including self-reported symptoms, wearable device outputs, and electronic health records at scale, although challenges with participant engagement, data completeness, and fraudulent enrollment require ongoing refinement (127).

Global federated learning networks coordinating collaborative model development across institutions and countries without centralizing sensitive patient data offer pragmatic pathways to address data scarcity and representativeness limitations currently constraining AI generalizability (100, 124). Federated architectures enable training algorithms on datasets distributed across low-income, middle-income, and high-income countries, systematically incorporating epidemiological diversity while respecting data sovereignty concerns and regulatory constraints that preclude international data sharing (100). The expansion of maternal health digital platforms integrating telemedicine consultations, mobile symptom tracking, automated risk alerts, and connections to community health workers creates comprehensive ecosystems that support women throughout pregnancy and postpartum (128). Recent implementations in Sub-Saharan Africa and South Asia demonstrate that culturally adapted digital platforms improve antenatal care access, emergency response coordination, and patient engagement, particularly when designed through community participatory approaches that center on end-user needs (129). However, sustainable scale-up requires addressing persistent challenges, including digital fragility from cybersecurity vulnerabilities, infrastructure limitations in connectivity and electricity, workforce digital literacy gaps, and financing mechanisms ensuring equitable access rather than perpetuating digital divide (128). The convergence of these technological trajectories with implementation science, health equity frameworks, and robust regulatory governance will ultimately determine whether artificial intelligence fulfills its transformative potential for maternal health or merely amplifies existing disparities under a veneer of technological sophistication.

## 12 Study limitations

This review has several limitations that should be acknowledged. First, as a structured narrative review rather than a formal systematic review or meta-analysis, the study did not employ quantitative pooled analyses or standardized risk-of-bias assessment tools across included studies. The reviewed literature was also methodologically heterogeneous, encompassing retrospective cohort studies, machine learning model development studies, systematic reviews, implementation analyses, and conceptual ethical frameworks, which limited direct comparison of predictive performance across studies. In addition, many included studies relied primarily on internally validated datasets, with comparatively limited prospective multicenter or external validation, potentially affecting the generalizability of reported performance metrics. Variability in outcome definitions, feature engineering strategies, dataset composition, and validation methodologies may further contribute to inconsistencies in reported AUROC values and translational readiness. Finally, although this review discusses explainability, fairness, and implementation considerations, evidence evaluating the long-term real-world clinical effectiveness and equity impact of AI-assisted maternal healthcare tools remains limited.

## 13 Conclusion

Artificial intelligence offers transformative potential to address the global burden of preventable maternal mortality through earlier risk detection, personalized antenatal surveillance, and resource optimization in under-resourced settings. However, this promise remains contingent on deliberately centering on health equity throughout development, validation, and deployment, rather than treating equity as an afterthought. The documented risks, including algorithmic bias perpetuating existing disparities, explainability challenges undermining clinical trust, regulatory vacuums creating accountability gaps, and infrastructure barriers limiting applicability in low-resource settings, demand proactive mitigation through inclusive dataset development, rigorous external validation, transparent performance reporting, and federated learning approaches that respect data sovereignty. Realizing AI's maternal health potential requires coordinated action-spanning algorithm design, clinical workflow integration, regulatory governance, and workforce capacity development, with implementation centered on the needs and preferences of pregnant women themselves. The window for responsible translation is now: failure to embed equity and accountability into AI systems will merely automate discrimination under a veneer of technological sophistication, ultimately widening rather than narrowing the chasm between promise and impact on maternal health globally.

## References

[1] Ward ZJ Atun R King G Sequeira Dmello B Goldie SJ. Simulation-based estimates and projections of global, regional and country-level maternal mortality by cause, 1990–2050. Nat Med. (2023) 29:1253. 10.1038/S41591-023-02310-X 37081226 PMC10202807

[2] Berhan Y Abeba S. Thirty years of united nations inter-agency working Group's Global, regional, and national maternal mortality estimates revisited. Int J MCH AIDS. (2024) 13:e004. 10.25259/IJMA_679 38694893 PMC11008585

[3] Moran AC Jolivet RR Chou D Dalglish SL Hill K Ramsey K. A common monitoring framework for ending preventable maternal mortality, 2015–2030: phase I of a multi-step process. BMC Pregnancy Childbirth. (2016) 16:250. 10.1186/S12884-016-1035-4 27565428 PMC5002107

[4] Stanton ME Kwast BE Shaver T McCallon B Koblinsky M. Beyond the safe motherhood initiative: accelerated action urgently needed to End preventable maternal mortality. Glob Health Sci Pract. (2018) 6:408. 10.9745/GHSP-D-18-00100 30093525 PMC6172132

[5] Von Dadelszen P Magee LA. Strategies to reduce the global burden of direct maternal deaths. Obstet Med. (2017) 10:5. 10.1177/1753495X16686287 28491124 PMC5405945

[6] Pandor A Daru J Hunt BJ Rooney G Hamilton J Clowes M. Risk assessment models for venous thromboembolism in pregnancy and in the puerperium: a systematic review. BMJ Open. (2022) 12:e065892. 10.1136/BMJOPEN-2022-065892 36223963 PMC9562726

[7] Kane SC Da Silva Costa F Brennecke S. First trimester biomarkers in the prediction of later pregnancy complications. Biomed Res Int. (2014) 2014:807196. 10.1155/2014/807196 24800250 PMC3988945

[8] Lee S Holden D Webb R Ayers S. Pregnancy related risk perception in pregnant women, midwives & doctors: a cross-sectional survey. BMC Pregnancy Childbirth. (2019) 19:335. 10.1186/S12884-019-2467-4 31558157 PMC6764151

[9] Cook K Perkins NJ Schisterman E Haneuse S. A multistate competing risks framework for preconception prediction of pregnancy outcomes. BMC Med Res Methodol. (2022) 22:156. 10.1186/S12874-022-01589-7 35637547 PMC9150288

[10] Islam MN Mustafina SN Mahmud T Khan NI. Machine learning to predict pregnancy outcomes: a systematic review, synthesizing framework and future research agenda. BMC Pregnancy Childbirth. (2022) 22:348. 10.1186/S12884-022-04594-2 35546393 PMC9097057

[11] Davidson L Boland MR. Towards deep phenotyping pregnancy: a systematic review on artificial intelligence and machine learning methods to improve pregnancy outcomes. Brief Bioinform. (2021) 22:bbaa369. 10.1093/BIB/BBAA369 33406530 PMC8424395

[12] Vivek Khanna V Chadaga K Sampathila N Prabhu S Chadaga PR Bhat D. Explainable artificial intelligence-driven gestational diabetes mellitus prediction using clinical and laboratory markers. Cogent Eng. (2024) 11:2330266. 10.1080/23311916.2024.2330266;ISSUE:ISSUE:DOI

[13] Pi X Wang J Chu L Zhang G Zhang W. Prediction of high-risk pregnancy based on machine learning algorithms. Sci Rep. (2025) 15:15561. 10.1038/S41598-025-00450-3 40319080 PMC12049423

[14] Liu X Chen Z Ji Y. Construction of the machine learning-based live birth prediction models for the first *in vitro* fertilization pregnant women. BMC Pregnancy Childbirth. (2023) 23:476. 10.1186/S12884-023-05775-3 37370040 PMC10294395

[15] Wang H Zhang Z Li H Li J Li H Liu M. A cost-effective machine learning-based method for preeclampsia risk assessment and driver genes discovery. Cell Biosci. (2023) 13:41. 10.1186/S13578-023-00991-Y 36849879 PMC9972636

[16] Espinosa C Becker M Marić I Wong RJ Shaw GM Gaudilliere B. Data-driven modeling of pregnancy-related complications. Trends Mol Med. (2021) 27:762. 10.1016/J.MOLMED.2021.01.007 33573911 PMC8324504

[17] Hussain SA Bresnahan M Zhuang J. The bias algorithm: how AI in healthcare exacerbates ethnic and racial disparities–a scoping review. Ethn Health. (2025) 30:197–214. 10.1080/13557858.2024.2422848;ISSUE:ISSUE:DOI 39488857

[18] Lin X Liang C Liu J Lyu T Ghumman N Campbell B. Artificial intelligence–augmented clinical decision support systems for pregnancy care: Systematic Review. J Med Internet Res. (2024) 26:e54737. 10.2196/54737 39283665 PMC11443205

[19] Celi LA Cellini J Charpignon ML Dee EC Dernoncourt F Eber R. Sources of bias in artificial intelligence that perpetuate healthcare disparities—a global review. PLOS Digit Health. (2022) 1:e0000022. 10.1371/JOURNAL.PDIG.0000022 36812532 PMC9931338

[20] Bolarinwa O Mohammed A Igharo V Shongwe S. Leveraging artificial intelligence for inclusive maternity care: enhancing access for mothers with disabilities in Africa. Women's Health. (2025) 21:17455057251326675. 10.1177/17455057251326675;PAGE:STRING:ARTICLE/CHAPTER PMC11910734 40089873

[21] Chen F Wang L Hong J Jiang J Zhou L. Unmasking bias in artificial intelligence: a systematic review of bias detection and mitigation strategies in electronic health record-based models. J Am Med Inform Assoc. (2024) 31:1172. 10.1093/JAMIA/OCAE060 38520723 PMC11031231

[22] Panch T Mattie H Atun R. Artificial intelligence and algorithmic bias: implications for health systems. J Glob Health. (2019) 9:020318. 10.7189/JOGH.09.020318 31788229 PMC6875681

[23] Ferryman K Cesare N Creary M Nsoesie EO. Racism is an ethical issue for healthcare artificial intelligence. Cell Rep Med. (2024) 5:101617. 10.1016/J.XCRM.2024.101617 38897175 PMC11228769

[24] Li T Xu M Wang Y Wang Y Tang H Duan H. Prediction model of preeclampsia using machine learning based methods: a population based cohort study in China. Front Endocrinol (Lausanne). (2024) 15:1345573. 10.3389/FENDO.2024.1345573/BIBTEX 38919479 PMC11198873

[25] Dkeen NOM Radwan MED Zumam IAA Mohamed NAAE Abdelmahmoud EMA Magboul NME. Artificial intelligence applications in obstetric risk prediction: a systematic review of machine learning models for preeclampsia. Cureus. (2025) 17(5):e83961. 10.7759/CUREUS.83961 40510095 PMC12158820

[26] Montgomery-Csobán T Kavanagh K Murray P Robertson C Barry SJE Vivian Ukah U. Machine learning-enabled maternal risk assessment for women with pre-eclampsia (the PIERS-ML model): a modelling study. Lancet Digit Health. (2024) 6:e238. 10.1016/S2589-7500(23)00267-4 38519152 PMC10983826

[27] Carbillon L. Commentary: AI-based preeclampsia detection and prediction with electrocardiogram data. Front Cardiovasc Med. (2024) 11:1437369. 10.3389/FCVM.2024.1437369 39139750 PMC11319159

[28] Xu Y Liu X Zhang Y Qi X Jin C Liang Z. Risk stratification and prediction of emergency delivery in early-onset preeclampsia using machine learning. BMC Med Inform Decis Mak. (2025) 25:414. 10.1186/S12911-025-03249-4 41199224 PMC12593787

[29] Pedersen L Mazur-Milecka M Ruminski J Wagner S. A review on machine learning deployment patterns and key features in the prediction of preeclampsia. Mach Learn Knowl Extr. (2024) 6:2515–69. 10.3390/MAKE6040123/S1

[30] Ding L Yin X Wen G Sun D Xian D Zhao Y. Prediction of preterm birth using machine learning: a comprehensive analysis based on large-scale preschool children survey data in Shenzhen of China. BMC Pregnancy Childbirth. (2024) 24:810. 10.1186/S12884-024-06980-4 39633287 PMC11616287

[31] Qian L Jia H Chang Z Hu Y Chen C Li X. Predicting the risk of preterm birth with machine learning and electronic health records in China. BMC Med Inform Decis Mak. (2025) 25:415. 10.1186/S12911-025-03254-7 41214653 PMC12604261

[32] Hassan A Nawaz S Tahira S Ahmed A. Preterm birth prediction using an explainable machine learning approach. *Artificial Intelligence and Applications*. Singapore: Bon View Publishing Pte Ltd (2025). 10.47852/BONVIEWAIA52024517

[33] Siargkas A Tsakiridis I Kappou D Mamopoulos A Papastefanou I Dagklis T. Machine learning models for the prediction of preterm birth at mid-gestation using individual characteristics and biophysical markers: a cohort study. Children. (2025) 12:1451. 10.3390/CHILDREN12111451 41300569 PMC12651481

[34] Teng X Liu M Wang Z Dong X. Machine learning prediction of preterm birth in women under 35 using routine biomarkers in a retrospective cohort study. Sci Rep. (2025) 15:10213. 10.1038/S41598-025-92814-Y 40133418 PMC11937320

[35] Seong D Espinosa C Aghaeepour N. Computational approaches for predicting preterm birth and newborn outcomes. Clin Perinatol. (2024) 51:461. 10.1016/J.CLP.2024.02.005 38705652 PMC11070639

[36] Khan W Zaki N Ghenimi N Ahmad A Bian J Masud MM. Predicting preterm birth using explainable machine learning in a prospective cohort of nulliparous and multiparous pregnant women. PLoS One. (2023) 18:e0293925. 10.1371/JOURNAL.PONE.0293925 38150456 PMC10752564

[37] Kolozali S White SL Norris S Fasli M Van Heerden A. Explainable early prediction of gestational diabetes biomarkers by combining medical background and wearable devices: a pilot study with a cohort group in South Africa. IEEE J Biomed Health Inform. (2024) 28:1860–71. 10.1109/JBHI.2024.3361505 38345955

[38] Zhou F Ran X Song F Wu Q Jia Y Liang Y. A stepwise prediction and interpretation of gestational diabetes mellitus: foster the practical application of machine learning in clinical decision. Heliyon. (2024) 10:e32709. 10.1016/J.HELIYON.2024.E32709 38975148 PMC11225730

[39] Cubillos G Monckeberg M Plaza A Morgan M Estevez PA Choolani M. Development of machine learning models to predict gestational diabetes risk in the first half of pregnancy. BMC Pregnancy Childbirth. (2023) 23:469. 10.1186/S12884-023-05766-4 37353749 PMC10288662

[40] Kumar M Ang LT Png H Ng M Tan K Loy SL. Automated machine learning (AutoML)-derived preconception predictive risk model to guide early intervention for gestational diabetes Mellitus. Int J Environ Res Public Health. (2022) 19:6792. 10.3390/IJERPH19116792/S1 35682375 PMC9180245

[41] Xing J Dong K Liu X Ma J Yuan E Zhang L. Enhancing gestational diabetes mellitus risk assessment and treatment through GDMPredictor: a machine learning approach. J Endocrinol Invest. (2024) 47:2351. 10.1007/S40618-024-02328-Z 38460091 PMC11369014

[42] Kirkwood JR Galloway N Lindsay RS Manataki A Wake DJ Reynolds RM. The use of machine learning to predict pharmacological therapy in gestational diabetes: a scoping review. Diabetic Med. (2025) 00:e70171. 10.1111/DME.70171;PAGEGROUP:STRING:PUBLICATION PMC12857867 41254474

[43] Liao LD Ferrara A Greenberg MB Ngo AL Feng J Zhang Z. Development and validation of prediction models for gestational diabetes treatment modality using supervised machine learning: a population-based cohort study. BMC Med. (2022) 20:307. 10.1186/S12916-022-02499-7 36104698 PMC9476287

[44] Pierucci UM Tonni G Pelizzo G Paraboschi I Werner H Ruano R. Artificial intelligence in fetal growth restriction management: a narrative review. J Clin Ultrasound. (2025) 53:825. 10.1002/JCU.23918 39887783 PMC12087706

[45] Rescinito R Ratti M Payedimarri AB Panella M. Prediction models for intrauterine growth restriction using artificial intelligence and machine learning: a systematic review and meta-analysis. Healthcare (Switzerland). (2023) 11:1617. 10.3390/HEALTHCARE11111617/S1 PMC10252230 37297757

[46] Zimmerman RM Hernandez EJ Yandell M Tristani-Firouzi M Silver RM Grobman W. AI-based analysis of fetal growth restriction in a prospective obstetric cohort quantifies compound risks for perinatal morbidity and mortality and identifies previously unrecognized high risk clinical scenarios. BMC Pregnancy Childbirth. (2025) 25:80. 10.1186/S12884-024-07095-6 39881241 PMC11780823

[47] Nogueira M Aparício SL Duarte I Silvestre M. Artificial Intelligence's Role in improving adverse pregnancy outcomes: a scoping review and consideration of ethical issues. J Clin Med. (2025) 14:3860. 10.3390/JCM14113860/S1 40507618 PMC12156818

[48] Lin YC Mallia D Clark-Sevilla AO Catto A Leshchenko A Yan Q. A comprehensive and bias-free machine learning approach for risk prediction of preeclampsia with severe features in a nulliparous study cohort. BMC Pregnancy Childbirth. (2024) 24:853. 10.1186/S12884-024-06988-W 39716098 PMC11667971

[49] Ahmed SAA Adam M Osman HMM Alqahtani NHF Gabreldaar AEM Abdalla MSH. Artificial intelligence for early detection of preeclampsia and gestational diabetes Mellitus: a systematic review of diagnostic performance. Cureus. (2025) 17(9):e92585. 10.7759/CUREUS.92585 41122552 PMC12536234

[50] Tudor CG Timofte DV Badulescu OV Ciobica A Ciobotariu T Mihalache AM. The accuracy of artificial intelligence to support multimodal management and prediction of gestational diabetes. Brain (Bacau). (2025) 16:325–30. 10.70594/BRAIN/16.4/20

[51] Susanu C Hărăbor A Vasilache IA Harabor V Călin AM. Predicting intra- and postpartum hemorrhage through artificial intelligence. Medicina (B Aires). (2024) 60:1604. 10.3390/MEDICINA60101604 PMC11509710 39459391

[52] Westcott JM Hughes F Liu W Grivainis M Hoskins I Fenyo D. Prediction of maternal hemorrhage using machine learning: retrospective cohort study. J Med Internet Res. (2022) 24:e34108. 10.2196/34108 35849436 PMC9345059

[53] Villalaín C Herraiz I Domínguez-Del Olmo P Angulo P Ayala JL Galindo A. Prediction of delivery within 7 days after diagnosis of early onset preeclampsia using machine-learning models. Front Cardiovasc Med. (2022) 9:910701. 10.3389/FCVM.2022.910701/FULL 35845049 PMC9283699

[54] Li H Chen M Zhang J. Research Progress on the Application of AI in Prenatal Diagnosis and Fetal Growth and Development. In: Proceedings of the 2024 3rd International Conference on Health Big Data and Intelligent Healthcare (ICHIH 2024). Piscataway, NJ: IEEE (2024). p. 348–51. 10.1109/ICHIH63459.2024.11064905

[55] Yaseen I Rather RA. A theoretical exploration of artificial Intelligence's Impact on feto-maternal health from conception to delivery. Int J Womens Health. (2024) 16:903–15. 10.2147/IJWH.S454127 38800118 PMC11128252

[56] Miskeen E Alfaifi J Alhuian DM Alghamdi M Alharthi MH Alshahrani NA. Prospective applications of artificial intelligence in fetal medicine: a scoping review of recent updates. Int J Gen Med. (2025) 18:237. 10.2147/IJGM.S490261 39834911 PMC11745059

[57] Pooh RK. First-trimester preterm preeclampsia prediction model for prevention with low-dose aspirin. J Obstet Gynaecol Res. (2024) 50:793–9. 10.1111/JOG.15908;REQUESTEDJOURNAL:JOURNAL:14470756;WGROUP:STRING:PUBLICATION 38366809

[58] Tousty P Fraszczyk-Tousty M Dzidek S Jasiak-Jóźwik H Michalczyk K Kwiatkowska E. Low-Dose aspirin after ASPRE—more questions than answers? Current international approach after PE screening in the first trimester. Biomedicines. (2023) 11:1495. 10.3390/BIOMEDICINES11061495 37371598 PMC10295279

[59] Hezelgrave NL Suff N Seed P Robinson V Carter J Watson H. Comparing cervical cerclage, pessary and vaginal progesterone for prevention of preterm birth in women with a short cervix (SuPPoRT): a multicentre randomised controlled trial. PLoS Med. (2024) 21:e1004427. 10.1371/JOURNAL.PMED.1004427 39012912 PMC11288449

[60] Bertini A Salas R Chabert S Sobrevia L Pardo F. Using machine learning to predict complications in pregnancy: a systematic review. Front Bioeng Biotechnol. (2022) 9:780389. 10.3389/FBIOE.2021.780389/FULL 35127665 PMC8807522

[61] Devi S Kushawaha A Shah D Gangarde R Suryavanshi MR Joshi C. Next-Gen midwifery support: designing an artificial intelligence (AI) enhanced Mobile app for pregnancy risk categorization and clinical decision support on maternal and neonatal outcomes. Birth. (2025) 53:388–98. 10.1111/BIRT.70037 41364741

[62] Tegenaw GS Amenu D Ketema G Verbeke F Cornelis J Jansen B. Evaluating a clinical decision support point of care instrument in low resource setting. BMC Med Inform Decis Mak. (2023) 23:51. 10.1186/S12911-023-02144-0 36998074 PMC10064719

[63] Khan M Khurshid M Vatsa M Singh R Duggal M Singh K. On AI approaches for promoting maternal and neonatal health in low resource settings: a review. Front Public Health. (2022) 10:880034. 10.3389/FPUBH.2022.880034 36249249 PMC9562034

[64] Amoakoh HB Klipstein-Grobusch K Grobbee DE Amoakoh-Coleman M Oduro-Mensah E Sarpong C. Using Mobile health to support clinical decision-making to improve maternal and neonatal health outcomes in Ghana: insights of frontline health worker information needs. JMIR Mhealth Uhealth. (2019) 7:e12879. 10.2196/12879 31127719 PMC6555115

[65] Amoakoh HB Klipstein-Grobusch K Ansah EK Grobbee DE Yveoo L Agyepong I. How and why front-line health workers (did not) use a multifaceted mHealth intervention to support maternal and neonatal healthcare decision-making in Ghana. BMJ Glob Health. (2019) 4:1153. 10.1136/BMJGH-2018-001153 PMC6441261 30997162

[66] Sisimayi C Visaya MV. Deployment of AI Models and a Mapper Algorithm to Enhance Clinical Decision-Making. 2024 International Conference on Sustainable Technology and Engineering, i-COSTE 2024 2024. 10.1109/I-COSTE63786.2024.11025118

[67] Iftikhar PM Kuijpers MV Khayyat A Iftikhar A DeGouvia De Sa M. Artificial intelligence: a new paradigm in obstetrics and gynecology research and clinical practice. Cureus. (2020) 12:e7124. 10.7759/CUREUS.7124 32257670 PMC7105008

[68] Davidson L Boland MR. Enabling pregnant women and their physicians to make informed medication decisions using artificial intelligence. J Pharmacokinet Pharmacodyn. (2020) 47:305. 10.1007/S10928-020-09685-1 32279157 PMC7473961

[69] Nagraj S Kennedy S Jha V Norton R Hinton L Billot L. A Mobile clinical decision support system for high-risk pregnant women in rural India (SMARThealth pregnancy): pilot cluster randomized controlled trial. JMIR Form Res. (2023) 7:e44362. 10.2196/44362 37471135 PMC10401191

[70] Marko JGO Neagu CD Anand PB. Examining inclusivity: the use of AI and diverse populations in health and social care: a systematic review. BMC Med Inform Decis Mak. (2025) 25:57. 10.1186/S12911-025-02884-1 39910518 PMC11796235

[71] Ahluwalia M Sehgal S Lee G Agu E Kpodonu J. Disparities in artificial intelligence–based tools among diverse minority populations: biases, barriers, and solutions. JACC: Advances. (2025) 4:101742. 10.1016/J.JACADV.2025.101742 40286381 PMC12103092

[72] Alderman JE Palmer J Laws E McCradden MD Ordish J Ghassemi M. Tackling algorithmic bias and promoting transparency in health datasets: the STANDING together consensus recommendations. Lancet Digit Health. (2024) 7:e64. 10.1016/S2589-7500(24)00224-3 39701919 PMC11668905

[73] Gu T Pan W Yu J Ji G Meng X Wang Y. Mitigating bias in AI mortality predictions for minority populations: a transfer learning approach. BMC Med Inform Decis Mak. (2025) 25:30. 10.1186/S12911-025-02862-7 39825353 PMC11742213

[74] Haider SA Borna S Gomez-Cabello CA Pressman SM Haider CR Forte AJ. The algorithmic divide: a systematic review on AI-driven racial disparities in healthcare. J Racial Ethn Health Disparities. (2024) 2024:1–30. 10.1007/S40615-024-02237-0 39695057

[75] Li DM Parikh S Costa A. A critical look into artificial intelligence and healthcare disparities. Front Artif Intell. (2025) 8:1545869. 10.3389/frai.2025.1545869 40115119 PMC11922879

[76] Hilling DE Ihaddouchen I Buijsman S Townsend R Gommers D van Genderen ME. The imperative of diversity and equity for the adoption of responsible AI in healthcare. Front Artif Intell. (2025) 8:1577529. 10.3389/FRAI.2025.1577529/BIBTEX 40309720 PMC12040885

[77] Ewals LJS Heesterbeek LJJ Yu B van der Wulp K Mavroeidis D Funk M. The impact of expectation management and model transparency on Radiologists' Trust and utilization of AI recommendations for lung nodule assessment on computed tomography: simulated use study. JMIR AI. (2024) 3:e52211. 10.2196/52211 38875574 PMC11041414

[78] Rosenbacke R Melhus Å McKee M Stuckler D. How explainable artificial intelligence can increase or decrease Clinicians' Trust in AI applications in health care: systematic Review. JMIR AI. (2024) 3:e53207. 10.2196/53207 39476365 PMC11561425

[79] Hasan R Dattana V Mahmood S Hussain S. Towards transparent diabetes prediction: combining AutoML and explainable AI for improved clinical insights. Information. (2025) 16:7. 10.3390/INFO16010007

[80] Matulionyte R Suero Molina E Di Ieva A. Neurosurgery, explainable AI, and legal liability. Adv Exp Med Biol. (2024) 1462:543–53. 10.1007/978-3-031-64892-2_34 39523289

[81] Ossa LA Starke G Lorenzini G Vogt JE Shaw DM Elger BS. Re-focusing explainability in medicine n.d. 10.1177/20552076221074488 PMC8841907 35173981

[82] Chandra J Kaur R Sahay R. Integrated Framework for Equitable Healthcare AI: Bias Mitigation, Community Participation, and Regulatory Governance. 2025 IEEE 14 International Conference on Communication Systems and Network Technologies, CSNT 2025 (2025):819–25. 10.1109/CSNT64827.2025.10968102

[83] Hasanzadeh F Josephson CB Waters G Adedinsewo D Azizi Z White JA. Bias recognition and mitigation strategies in artificial intelligence healthcare applications. npj Digit Med. (2025) 8:154. 10.1038/s41746-025-01503-7 40069303 PMC11897215

[84] Dehghani F Paiva P Malik N Lin J Bayat S Bento M. Navigating Fairness in Healthcare: A Comparative Analysis of Single-Stage and Multi-Stage Bias Mitigation Strategies. 2025 IEEE International Conference on Software Analysis, Evolution and Reengineering - Companion (SANER-C) (2025):41–8. 10.1109/SANER-C66551.2025.00013

[85] Gichoya JW Thomas K Celi LA Safdar N Banerjee I Banja JD. AI Pitfalls and what not to do: mitigating bias in AI. Br J Radiol. (2023) 96:20230023. 10.1259/BJR.20230023 37698583 PMC10546443

[86] Mullankandy S Mukherjee S Ingole BS. Applications of AI in Electronic Health Records, Challenges, and Mitigation Strategies. 2024 International Conference on Computer and Applications, ICCA 2024 (2024). 10.1109/ICCA62237.2024.10927863

[87] Thomasian NM Eickhoff C Adashi EY. Advancing health equity with artificial intelligence. J Public Health Policy. (2021) 42:602. 10.1057/S41271-021-00319-5 34811466 PMC8607970

[88] Ranjbar A Montazeri F Ghamsari SR Mehrnoush V Roozbeh N Darsareh F. Machine learning models for predicting preeclampsia: a systematic review. BMC Pregnancy Childbirth. (2024) 24:6. 10.1186/S12884-023-06220-1 38166801 PMC10759509

[89] Liang Y Dai A Luo M Zheng Z Shen J Su Y. Predictive performance of artificial intelligence algorithms for gestational diabetes Mellitus in pregnant women: systematic review and meta-analysis. J Med Internet Res. (2026) 28:e79729. 10.2196/79729 41616232 PMC12858046

[90] Montgomery-Csobán T Kavanagh K Murray P Robertson C Barry SJE Vivian Ukah U. Machine learning-enabled maternal risk assessment for women with pre-eclampsia (the PIERS-ML model): a modelling study. Lancet Digit Health. (2024) 6:e238–50. 10.1016/S2589-7500(23)00267-4 38519152 PMC10983826

[91] Ji Q Wang M. AI-driven high-risk pregnancy prediction: balancing early detection, anxiety, and discrimination in digital public health. Front Public Health. (2026) 14:1752484. 10.3389/FPUBH.2026.1752484/TEXT 41971288 PMC13062171

[92] Ahmed MI Spooner B Isherwood J Lane M Orrock E Dennison A. A Systematic Review of the Barriers to the Implementation of Artificial Intelligence in Healthcare Introduction And Background (2023). 10.7759/cureus.46454 PMC10623210 37927664

[93] Nair M Svedberg P Larsson I Nygren JM. A comprehensive overview of barriers and strategies for AI implementation in healthcare: mixed-method design. PLoS One. (2024) 19:e0305949. 10.1371/JOURNAL.PONE.0305949 39121051 PMC11315296

[94] López DM Rico-Olarte C Blobel B Hullin C. Challenges and solutions for transforming health ecosystems in low- and middle-income countries through artificial intelligence. Front Med (Lausanne). (2022) 9:958097. 10.3389/FMED.2022.958097 36530888 PMC9755337

[95] Kaushik A Barcellona C Mandyam NK Tan SY Tromp J. Challenges and opportunities for data sharing related to artificial intelligence tools in health care in low- and middle-income countries: Systematic Review and Case Study From Thailand. J Med Internet Res. (2025) 27:e58338. 10.2196/58338 39903508 PMC11836587

[96] Oduoye MO Fatima E Muzammil MA Dave T Irfan H Fariha FNU. Impacts of the advancement in artificial intelligence on laboratory medicine in low- and middle-income countries: challenges and recommendations—a literature review. Health Sci Rep. (2024) 7:e1794. 10.1002/HSR2.1794;JOURNAL:JOURNAL:23988835 38186931 PMC10766873

[97] Xie H Dai X Xie J Lei S Zeng J Yang J. Artificial intelligence adoption in surgery: cognition, usage patterns and implementation barriers of DeepSeek among healthcare professionals in China's Tertiary hospitals. J Multidiscip Healthc. (2025) 18:7719–37. 10.2147/JMDH.S538723 41334158 PMC12666401

[98] Tan Y Peng Y Guo L Liu D Luo Y. Cost-effectiveness analysis of AI-based image quality control for perinatal ultrasound screening. BMC Med Educ. (2024) 24:1437. 10.1186/S12909-024-06477-W 39696216 PMC11654377

[99] Brandão M Mendes F Martins M Cardoso P Macedo G Mascarenhas T. Revolutionizing Women's Health: a comprehensive review of artificial intelligence advancements in gynecology. J Clin Med. (2024) 13:1061. 10.3390/JCM13041061 38398374 PMC10889757

[100] Rahimi SA Shrivastava R Brown-Johnson A Caidor P Davies C Janati AI. EDAI Framework for integrating equity, diversity, and inclusion throughout the lifecycle of AI to improve health and oral health care: qualitative study. J Med Internet Res. (2024) 26:e63356. 10.2196/63356 39546793 PMC11607576

[101] De Hond AAH Van Buchem MM Hernandez-Boussard T. Picture a data scientist: a call to action for increasing diversity, equity, and inclusion in the age of AI. J Am Med Inform Assoc. (2022) 29:2178. 10.1093/JAMIA/OCAC156 36048021 PMC9667164

[102] Yuan H. Toward real-world deployment of machine learning for health care: external validation, continual monitoring, and randomized clinical trials. Health Care Science. (2024) 3:360. 10.1002/HCS2.114 39479276 PMC11520244

[103] Alfaraj SA Kist JM Groenwold RHH Spruit M Mook-Kanamori D Vos RC. External validation of SCORE2-diabetes in The Netherlands across various socioeconomic levels in native-Dutch and non-Dutch populations. Eur J Prev Cardiol. (2025) 32:555–63. 10.1093/EURJPC/ZWAE354 39485827

[104] Dreisbach C Barcelona V Turchioe MR Bernstein S Erickson E. Application of predictive analytics in pregnancy, birth, and postpartum nursing care. MCN Am J Matern Child Nurs. (2025) 50:66–77. 10.1097/NMC.0000000000001082 39724545 PMC12212750

[105] Park SH Suh CH. Reporting guidelines for artificial intelligence studies in healthcare (for both conventional and large language models): what's New in 2024. Korean J Radiol. (2024) 25:687. 10.3348/KJR.2024.0598 39028011 PMC11306008

[106] Newton N Bamgboje-Ayodele A Forsyth R Tariq A Baysari MT. A systematic review of clinicians' Acceptance and use of clinical decision support systems over time. NPJ Digit Med. (2025) 8:309. 10.1038/s41746-025-01662-7 40419669 PMC12106704

[107] Nadarzynski T Knights N Husbands D Graham CA Llewellyn CD Buchanan T. Achieving health equity through conversational AI: a roadmap for design and implementation of inclusive chatbots in healthcare. PLOS Digit Health. (2024) 3:e0000492. 10.1371/JOURNAL.PDIG.0000492 38696359 PMC11065243

[108] Yang YT Ricciardi R. Regulating AI in nursing and healthcare: ensuring safety, equity, and accessibility in the era of federal innovation policy. Policy Polit Nurs Pract. (2025) 27:17–25. 10.1177/15271544251381228;REQUESTEDJOURNAL:JOURNAL:PPNA;WEBSITE:WEBSITE:SAGE;WGROUP:STRING:PUBLICATION 41032683

[109] Giaxi P Vivilaki V Sarella A Gourounti K. Artificial intelligence in midwifery: a scoping review of current applications, future prospects, and Midwives' Perspectives. Healthcare. (2025) 13:942. 10.3390/HEALTHCARE13080942 40281891 PMC12026693

[110] Laguitan RVM Bernardino GD. Can AI bridge or widen maternal health inequities? Public Health Challenges. (2025) 4:e70119. 10.1002/PUH2.70119 40978155 PMC12445195

[111] Fee N Glover LE Bauman R Crosby DA. Patient perceptions on the use of artificial intelligence (AI) in fertility treatment. Hum Fertil. (2025) 28(1):2591161. 10.1080/14647273.2025.2591161 41320951

[112] Foresman G Biro J Tran A MacRae K Kazi S Schubel L. Patient perspectives on artificial intelligence in health care: focus group study for diagnostic communication and tool implementation. J Particip Med. (2025) 17:e69564. 10.2196/69564 40705399 PMC12288699

[113] Wang Y Ma Z. Ethical and legal challenges of medical AI on informed consent: china as an example. Dev World Bioeth. (2025) 25:46–54. 10.1111/DEWB.12442 38240080

[114] Alami H Alami H Rivard L Rivard L Lehoux P Lehoux P. Artificial intelligence in health care: laying the foundation for responsible, sustainable, and inclusive innovation in low- and middle-income countries. Global Health. (2020) 16:52. 10.1186/S12992-020-00584-1 32580741 PMC7315549

[115] Purkayastha S Bhagwat H Sunchu K Hoilett O Odari E Thuo R. Development of AI-integrated infrastructure with biomedical device and mobile app for neonatal vital monitoring during and in between kangaroo care sessions. 2025 47 Annual International Conference of the IEEE Engineering in Medicine and Biology Society (EMBC). (2025) 1–7. 10.1109/EMBC58623.2025.11251596 41336822

[116] Baxter MS White A Lahti M Murto T Evans J. Machine learning in a time of COVID-19 - can machine learning support community health workers (CHWs) in low and middle income countries (LMICs) in the new normal? J Glob Health. (2021) 11:03017. 10.7189/JOGH.11.03017 33643627 PMC7898557

[117] Elangovan R Elangovan K Rani Sethuraj J Ccrn BR Krishnan E Tech M. OR33-04 A simple Mobile artificial intelligence retina tracker (SMART) powered by efficient deep learning models for diagnosis and prognosis of diabetic retinopathy. J Endocr Soc. (2025) 9(Supplement_1):bvaf149–1034. 10.1210/JENDSO/BVAF149.1034

[118] Mallon O Lippert F Pilot E. Artificial intelligence in prehospital emergency care systems in low- and middle-income countries: cure or curiosity? Insights from a qualitative study. Front Public Health. (2025) 13:1632029. 10.3389/FPUBH.2025.1632029/BIBTEX 41103483 PMC12521113

[119] Moodley Y Asare K Tanser F Tomita A. Maternal exposure to heat and its association with miscarriage in rural KwaZulu-natal, South Africa: a population-based cohort study. Women's Health. (2024) 20:17455057241259171. 10.1177/17455057241259171;ISSUE:ISSUE:DOI PMC11282531 39066467

[120] Papadiochou A Diamanti A Metallinou D Georgakopoulou VE Taskou C Kagkouras I. Impact of climate change on reproductive health and pregnancy outcomes: a systematic review. Cureus. (2024) 16:e68221. 10.7759/CUREUS.68221 39347228 PMC11439441

[121] Zakir Hussain I Oetomo A Kaur J Morita PP. Next-generation public health surveillance: extreme heat event prediction and monitoring system. Eur J Public Health. (2024) 34(Supplement_3):ckae144–1045. 10.1093/EURPUB/CKAE144.1045

[122] Johnston C Nashihah P Purnami CT Martini M. The surveillance of emergent threats to maternal and newborn health in Indonesia: a scoping review. Journal of Public Health for Tropical and Coastal Region. (2024) 7:209–21. 10.14710/JPHTCR.V7I3.24292

[123] Ciecierski-Holmes T Singh R Axt M Brenner S Barteit S. Artificial intelligence for strengthening healthcare systems in low- and middle-income countries: a systematic scoping review. NPJ Digit Med. (2022) 5:162. 10.1038/S41746-022-00700-Y 36307479 PMC9614192

[124] van Hilten A van Rooij J Heijmans BT ‘t Hoen PAC van Meurs J Jansen R. Phenotype prediction using biologically interpretable neural networks on multi-cohort multi-omics data. NPJ Syst Biol Appl. (2024) 10:81. 10.1038/S41540-024-00405-W 39095438 PMC11297229

[125] Marić I Stevenson DK Aghaeepour N Gaudillière B Wong RJ Angst MS. Predicting preterm birth using proteomics. Clin Perinatol. (2024) 51:391. 10.1016/J.CLP.2024.02.011 38705648 PMC11186213

[126] Georgieva A Abry P Nunes I Frasch MG. Editorial: fetal-maternal monitoring in the age of artificial intelligence and computer-aided decision support: a multidisciplinary perspective. Front Pediatr. (2022) 10:1007799. 10.3389/FPED.2022.1007799 36133792 PMC9483201

[127] Ajayi T Kueper J Ariniello L Ho D Delgado F Beal M. Digital health platform for maternal health: design, recruitment strategies, and lessons learned from the PowerMom observational cohort study. JMIR Form Res. (2025) 9:e70149. 10.2196/70149 40194282 PMC12012398

[128] Mohamed H Ismail A Sutan R Rahman RA Juval K. A scoping review of digital technologies in antenatal care: recent progress and applications of digital technologies. BMC Pregnancy Childbirth. (2025) 25:153. 10.1186/S12884-025-07209-8 39948493 PMC11827299

[129] Nkangu M Njoache MN Obegu P Wanda F Ngo NV Fantaye A. Developing the BornFyne prenatal management system version 2.0: a mixed method community participatory approach to digital health for reproductive maternal health. Oxford Open Digital Health. (2024) 2:oqae012. 10.1093/OODH/OQAE012. 40230970 PMC11932395

